# A new short-faced archosauriform from the Upper Triassic *Placerias*/Downs’ quarry complex, Arizona, USA, expands the morphological diversity of the Triassic archosauriform radiation

**DOI:** 10.1007/s00114-021-01733-1

**Published:** 2021-07-02

**Authors:** Andrew B. Heckert, Sterling J. Nesbitt, Michelle R. Stocker, Vince P. Schneider, Devin K. Hoffman, Brian W. Zimmer

**Affiliations:** 1 Department of Geological & Environmental Sciences, Appalachian State University, ASU Box 32067, Boone, NC 28607 USA; 2 North Carolina Museum of Natural Sciences, Raleigh, NC USA; 3 Virginia Polytechnic Institute and State University, Blacksburg, VA USA

**Keywords:** Crocodylomorpha, Convergent evolution, Norian, Jaw, Diversity, Archosauriformes

## Abstract

The *Placerias*/Downs’ Quarry complex in eastern Arizona, USA, is the most diverse Upper Triassic vertebrate locality known. We report a new short-faced archosauriform, *Syntomiprosopus sucherorum* gen. et sp. nov., represented by four incomplete mandibles, that expands that diversity with a morphology unique among Late Triassic archosauriforms. The most distinctive feature of *Syntomiprosopus* gen. nov. is its anteroposteriorly short, robust mandible with 3–4 anterior, a larger caniniform, and 1–3 “postcanine” alveoli. The size and shape of the alveoli and the preserved tips of replacement teeth preclude assignment to any taxon known only from teeth. Additional autapomorphies of *S. sucherorum* gen. et sp. nov. include a large fossa associated with the mandibular fenestra, an interdigitating suture of the surangular with the dentary, fine texture ornamenting the medial surface of the splenial, and a surangular ridge that completes a 90° arc. The external surfaces of the mandibles bear shallow, densely packed, irregular, fine pits and narrow, arcuate grooves. This combination of character states allows an archosauriform assignment; however, an associated and similarly sized braincase indicates that *Syntomiprosopus* n. gen. may represent previously unsampled disparity in early-diverging crocodylomorphs. The *Placerias* Quarry is Adamanian (Norian, maximum depositional age ~219 Ma), and this specimen appears to be an early example of shortening of the skull, which occurs later in diverse archosaur lineages, including the Late Cretaceous crocodyliform *Simosuchus*. This is another case where Triassic archosauriforms occupied morphospace converged upon by other archosaurs later in the Mesozoic and further demonstrates that even well-sampled localities can yield new taxa.

**Supplementary Information:**

The online version contains supplementary material available at 10.1007/s00114-021-01733-1.

## Introduction

Birds and crocodylians represent the two surviving lineages of the spectacularly diverse clade Archosauria and its larger group Archosauromorpha, which includes non-avian dinosaurs, pterosaurs, and a variety of other extinct taxa representing a wide range of morphologies that occupied diverse ecological niches. Most, if not all, of these lineages have their roots in the Triassic Period, when crown-group Archosauria emerged as part of the larger radiation of archosauromorph reptiles. That clade rose from relative rarity in the Permian Period to dominate the mid- and large-body size (>10 kg) guilds in terrestrial and freshwater aquatic realms by the end of the Triassic (e.g., Fraser 2006; Sues and Fraser 2010; Nesbitt et al. 2013; Ezcurra et al. 2014). Several consistent themes have emerged from the past two decades of discovery and study of this evolutionary event: (1) the origins of many crown-group archosaurs have been pulled down into the Middle, or even Early, Triassic (e.g., Brusatte et al. 2010; Nesbitt 2011; Nesbitt et al. 2017a, b); (2) conversely, many early-diverging archosauromorphs and archosauriforms are known to have persisted into the Late Triassic (e.g., doswelliids, Weems 1980; Heckert et al. 2012; Sues et al. 2013; Wynd et al. 2020; *Vancleavea*, Nesbitt et al. 2009; tanystropheids; Olsen 1979; Pritchard et al. 2015); and (3) the radiation of archosauromorphs includes many examples of convergent evolution, where Triassic taxa established the bounds of a morphospace that was only much later explored by dinosaurs and other taxa (e.g., Nesbitt and Norell 2006; Stocker et al. 2016; Sengupta et al. 2017). Though many of the new discoveries that define these trends have resulted from the discovery of fossils from previously unexplored regions, it is clear that even well-studied stratigraphic intervals and localities are continuing to yield unexpected new taxa. Examples include the superficially ornithomimid-like *Effigia okeeffeae* from the Whitaker (*Coelophysis*) Quarry at Ghost Ranch, a site originally discovered in 1947 (Nesbitt and Norell 2006; Nesbitt 2007), and *Triopticus primus* from the Otis Chalk localities excavated in the late 1930s and 1940s (Stocker 2013; Stocker et al. 2016). However, the most diverse of these historic localities in the American Southwest is the *Placerias*/Downs’ Quarry complex in northeastern Arizona.

**Fig. 1:**
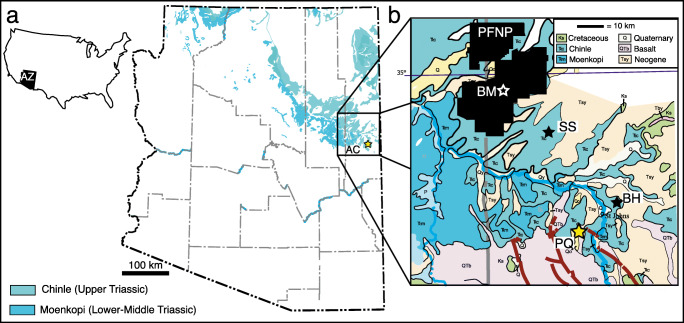
Index maps showing the geographic position of the *Placerias* quarry (PQ, yellow star) in **a** the USA and Arizona and **b** on a generalized geologic map of east-central Arizona (modified from Richard et al. 2002) including the most prolific Triassic localities in east-central Arizona. AC Apache County, BH Blue Hills, BM, Blue Mesa, PFNP Petrified Forest National Park (showing administrative boundary), SS Stinking Springs Mountain

The *Placerias* Quarry, (Fig. 1a, b) was first excavated in the 1930s, and by the 1990s this locality and the nearby (~72 m distant but almost certainly with 3 m stratigraphically) Downs’ Quarry were easily the most diverse nonmarine Triassic tetrapod assemblage known (Lucas et al. 1992; Kaye and Padian 1994; Long and Murry 1995). Reanalysis of fossils from these quarries continues to yield new taxa (e.g., Sues 1996; Hunt et al. 1998; Stocker et al. 2019). Though the assemblage as a whole merits reanalysis in light of these publications and would benefit from a more explicitly apomorphy-based approach, it is still clear that there are dozens of taxa represented. In 2010, we reopened excavations at the *Placerias*/Downs’ Quarry complex, recovering fossils that represent both previously known taxa and at least one new taxon; the latter is the focus of this contribution.

In this paper, we document a new taxon, *Syntomiprosopus sucherorum* gen. et sp. nov., that is morphologically distinct from previously known Triassic archosauromorphs and is simultaneously superficially convergent with later taxa such as the aberrant notosuchian crocodylomorph *Simosuchus clarki* (Buckley et al. 2000; see also Kley et al. 2010). Importantly, this new discovery comes from the *Placerias*/Downs’ Quarry complex, one of the longest known, most extensively worked, and diverse Upper Triassic tetrapod localities in the world, demonstrating that even in well-known localities, disparate new taxa remain to be discovered.

We provide details of the materials used to collect, prepare, image, and visualize the fossils described here in the Electronic Supplementary Material as Online Resource 1.

Institutional abbreviations: ASU, Appalachian State University, Boone, North Carolina, USA; CM, Carnegie Museum of Natural History, Pittsburgh, Pennsylvania, USA; IVPP, Institute of Vertebrate Paleontology and Paleoanthropology, Chinese Academy of Sciences, Beijing, China; MNA, Museum of Northern Arizona, Flagstaff, Arizona, USA; NCSM, North Carolina Museum of Natural Sciences, Raleigh, North Carolina, USA; TTUP, Texas Tech University Paleontology Collections, Lubbock, Texas, USA; UCMP, University of California Museum of Paleontology, Berkeley, California, USA.

## History of study

The *Placerias* Quarry was discovered in 1930 by C.L. Camp, and the history of excavation at the quarry is well documented (e.g., Camp and Welles 1956; Long et al. 1989; Long and Murry 1995; Parker 2018). The vast majority of the known macrovertebrate assemblage is based on UCMP collections made by Camp’s crews in the 1930s (e.g., Long and Murry 1995). In 1978–1979, the MNA excavated the *Placerias* Quarry and a nearby, stratigraphically higher (≤3 m) locality, the Downs’ Quarry, and they were the first to employ screenwashing techniques at these sites. The MNA collections are the basis for the bulk of the microvertebrate diversity from this locality (Jacobs and Murry 1980; Murry 1987; Kaye and Padian 1994). Some fossils and more modern taphonomic data were collected when the UCMP re-opened the site in 1989, 1990, and 1992 (Fiorillo and Padian 1993; Fiorillo et al. 2000). From 2010 to 2015, crews from the NCSM and ASU worked both the *Placerias* (2010–2011) and Downs’ (2010–2015) quarries, primarily targeting larger vertebrates but employing microvertebrate techniques as well. Personnel from Virginia Tech assisted in 2014–2015. All specimens from these recent excavations are housed at the NCSM.

## Stratigraphy and age

**Fig. 2:**
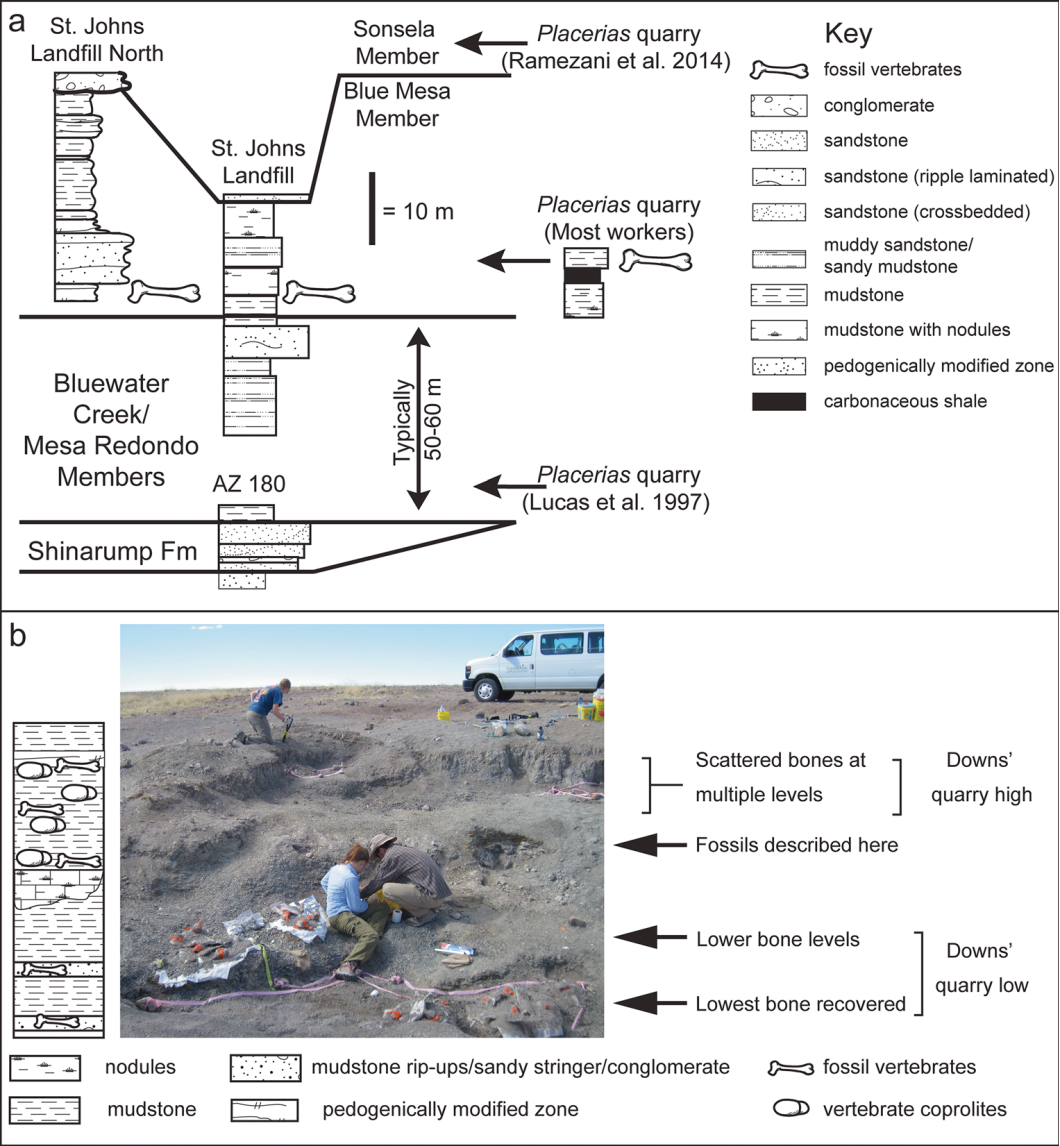
**a** Generalized stratigraphic sections of Triassic strata in the region based on Lucas et al. (1997), Heckert and Lucas (1997, 2003) and detailed stratigraphy of fossil occurrences at the Downs’ Quarry. The *Placerias* Quarry has traditionally been interpreted as occurring in strata currently assigned to the Blue Mesa Member. Lucas et al. (1997) thought it stratigraphically lower, and Ramezani et al. (2014) correlated it with the Sonsela Member in the PFNP. **b** Field photograph of excavations at the Downs’ Quarry with stratigraphic interpretation (left) and distribution of vertebrate fossils (right). Almost all fossils were recovered from either above (Downs' quarry high) or below (Downs' quarry low) the horizon that yielded the fossils described here

The stratigraphic position of the *Placerias*/Downs’ Quarry complex has long remained enigmatic (see summary by Lucas et al. 1997). The quarry complex is located in an area with relatively poor rock exposure and subdued topographic relief that is also near the southern margin of the Chinle outcrop belt, generally (Fig. 1a). Here, the Chinle section is considerably thinner than it is farther to the north, such as at Petrified Forest National Park. This is probably because it is condensed, nearing its southern depositional limit, and is locally truncated by one or more post-depositional erosional events. Recently, Ramezani et al. (2014) obtained a maximum depositional age (MDA) of 219.39 ± 0.16 Ma for detrital zircons they recovered while visiting the NCSM-ASU excavation of the quarry complex in 2013. This age agrees well with numerical estimates obtained from the base of the Blue Mesa Member elsewhere (Heckert et al. 2009; Irmis et al. 2011), although Ramezani et al. (2014) correlated this horizon to the Jasper Forest Bed of the Sonsela Member (Fig. 2a), which yielded a similar maximum depositional age in PEFO, a correlation followed by Parker (2018), but not by later workers (e.g., Kent et al. 2019; Marsh et al. 2019; Rasmussen et al. 2020), who considered the MDA of the Sonsela to be younger.

In spite of the importance of the *Placerias*/Downs’ quarries, including detailed work on assemblages of microvertebrates (Kaye and Padian 1994) and taphonomy (Fiorillo and Padian 1993; Fiorillo et al. 2000), there is no published stratigraphic column of the quarry complex. Camp and Welles (1956) described two fossiliferous levels at the *Placerias* Quarry proper, whereas Jacobs and Murry (1980) described fossils throughout the section, including above and below the horizons indicated by Camp and Welles (1956). Figure 2b shows our interpretations of the local stratigraphy, acknowledging that few, if any, of the horizons are truly planar. The NCSM-ASU excavations recovered fossils from two primary stratigraphic levels, termed “Downs’ Quarry low” and “Downs’ Quarry high” in the field to discriminate position relative to a persistent carbonate bed (Fig. 2b). At the NCSM-ASU excavations, the stratigraphically lowest horizon includes abundant bones, principally osteoderms and other postcrania of the aetosaur *Desmatosuchus* (e.g., NCSM 26643, a large lateral cervical osteoderm), as well as locally rich pockets of intraformational conglomerate with abundant microvertebrates (Fig. 2b). Approximately a meter above this lowest level is another horizon that is lithologically similar but contains much less bone and is locally barren, and on the east side of the excavations this interval is marked by a thick pedogenic(?) carbonate. Immediately above this level fossils are uncommon but include isolated phytosaur postcrania and much, if not all, of the new taxon described here, often associated with coprolites. The strata above this horizon (“Downs’ Quarry high”) correspond to the original Downs’ Quarry as excavated by the MNA and yield more scattered bones of some larger and diverse smaller vertebrates, many of them weathered and broken prior to burial in the Triassic Period, as well as abundant coprolites. Additional details are provided in the Electronic Supplementary Material as Online Resource 1.

## Results

### Systematic paleontology

ARCHOSAUROMORPHA von Huene 1946 sensu Benton 1985

ARCHOSAURIFORMES Gauthier et al. 1988

?CROCODYLOMORPHA Walker 1968 sensu Sereno et al. 2005

*Syntomiprosopus* gen. nov.

*Syntomiprosopus sucherorum* gen. et sp. nov.

**Fig. 3:**
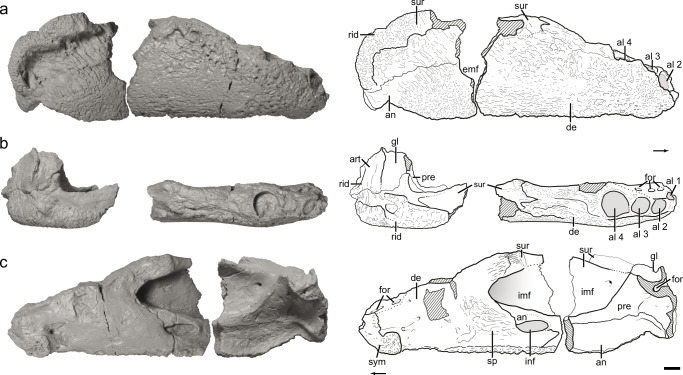
Holotype right mandible (NCSM 29059-29060) of *Syntomiprosopus sucherorum* gen. et sp. nov., with interpretive sketches in **a** lateral, **b** dorsal, and **c** medial views. NCSM 29059 is the anterior element, NCSM 29060 posterior. Abbreviations: al alveolus (numerals refer to tooth positions), an angular, art articular, d dentary, emf external mandibular fenestra, for foramen, g glenoid, imf internal mandibular fenestra, inf inframeckelian fenestra, pre prearticular, rid ridge, sp splenial, sur surangular. Cross hatching = broken/missing bone. Arrows indicate anterior direction. Scale bars = 1 cm

**Fig. 4:**
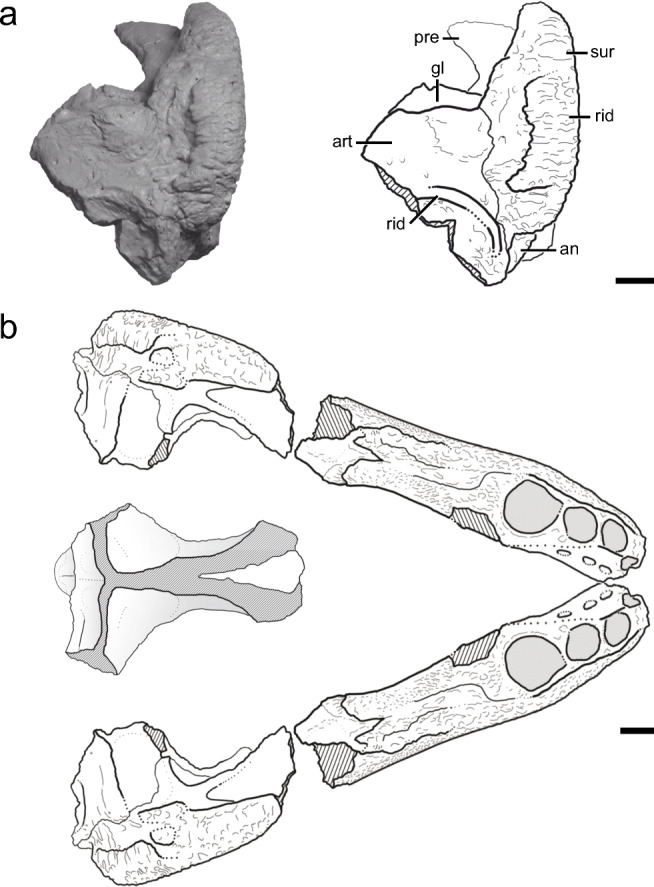
Illustration of the holotype right mandible of *Syntomiprosopus sucherorum* gen. et sp. nov. (**a**) and reconstruction of the jaws and posterior skull (**b**). **a** Holotype right mandible (NCSM 2906) in posterior view. **b** Reconstruction of the lower jaws based on mirroring the holotype jaw (NCSM 29069-29060) including the tentatively referred posterior skull and braincase (NCSM 27679) in dorsal view. The posterior portion of the skull and braincase (NCSM 27679) is illustrated in greater detail in Figure 8. Abbreviations as in Fig. 3. Scale bars = 1 cm

**Fig. 5.**
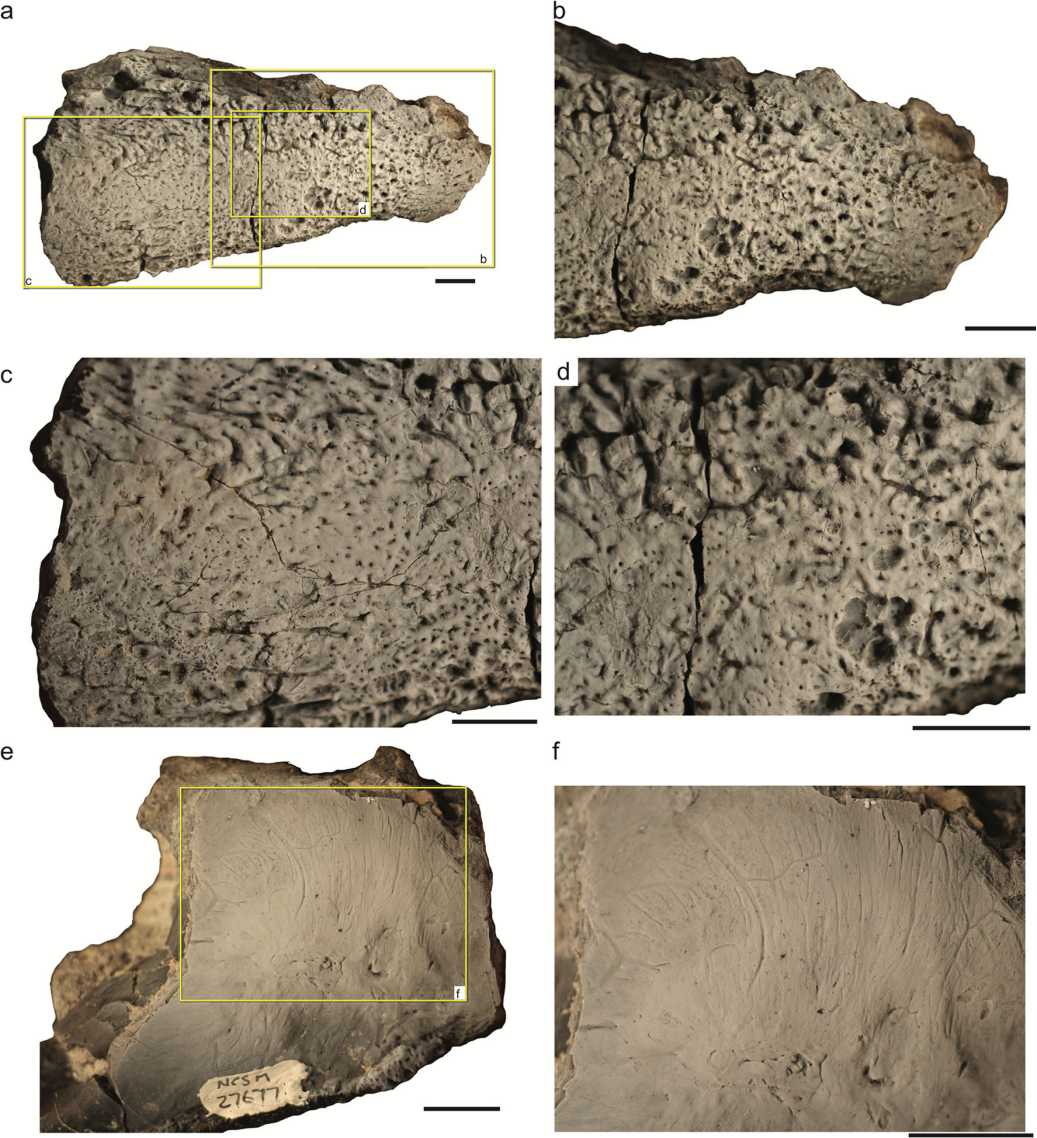
Holotype (NCSM 29059, **a**–**d**) and paratype (NCSM 27677, **e–f**) specimens of *Syntomiprosopus sucherorum* gen. et. sp. nov. showing the unique texture on the mandible. **a**–**d** Overview (**a**) and details (**b**–**d**) of holotype right anterior mandible (NCSM 29059) in lateral view with boxes in (**a**) delimiting close-up views of (**b**–**d**). **e**–**f** Paratype left posterior mandible (NMCSM 27677) in medial view with box in (**e**) delimiting close-up view of (**f**). Scale bars = 1 cm

**Fig. 6:**
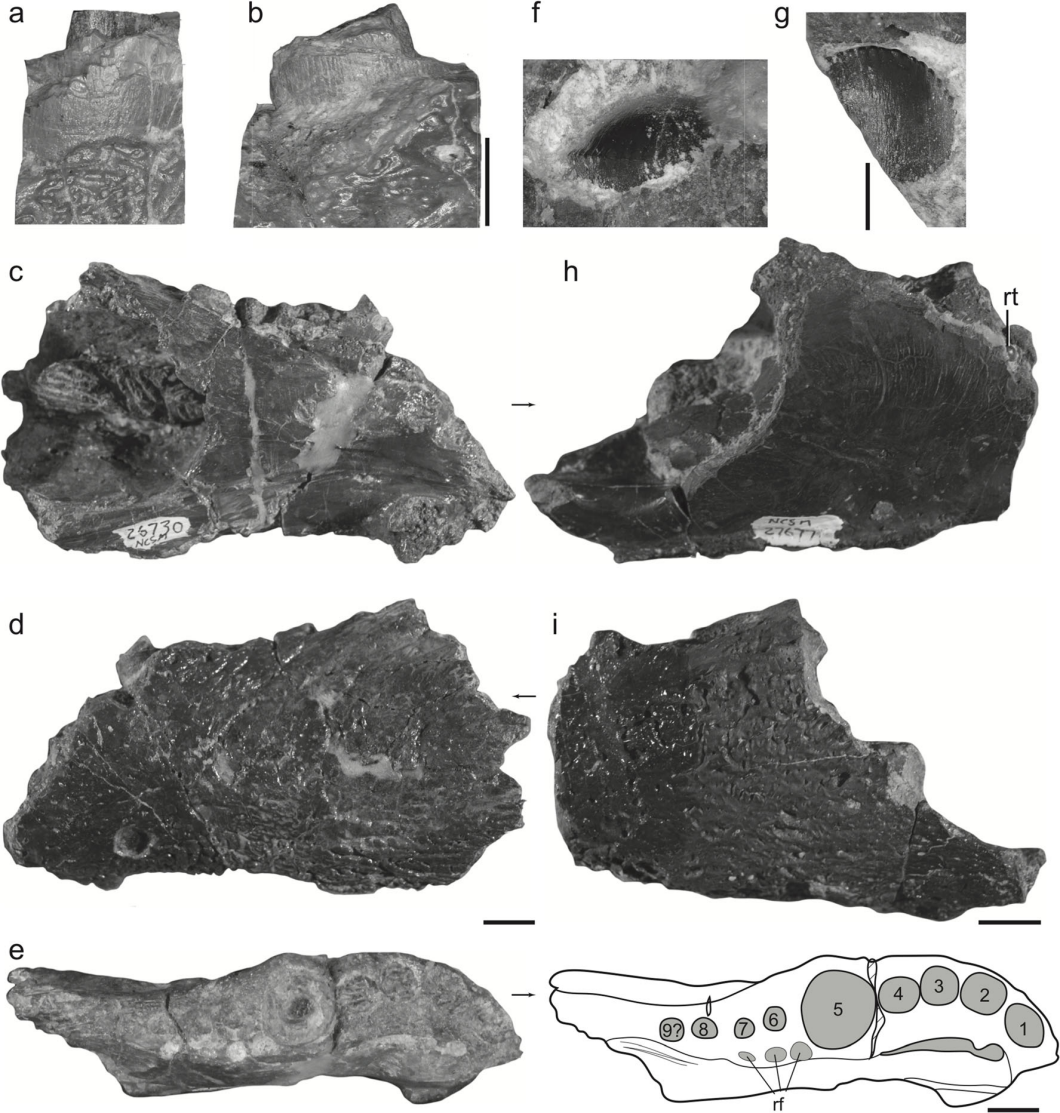
Tooth-bearing elements referred to *Syntomiprosopus sucherorum* gen. et sp. nov. **a**–**e** Left jaw with fractured caniniform tooth (NCSM 26730). **a** Base of fractured caniniform tooth in labial view, **b** base of fractured caniniform tooth in lingual view, **c** anterior portion of jaw in medial view, **d** anterior portion of jaw in lateral view, and **e** anterior portion of jaw in dorsal (occlusal) view (right) and with labels (left). **f**–**i** Incomplete left jaw including replacement tooth (NCSM 27677). **f** Replacement tooth labial to 6th socket position in occlusal view, **g** replacement tooth in labial view, **h** jaw fragment in medial view, and **i** jaw fragment in lateral view. Numerals refer to tooth positions. rf replacement foramina, rt replacement tooth. Scales = 5 mm (**a**–**b**), 2 cm (**c**–**e**, **h**–**i**), and 1 mm (**f**–**g**)

**Fig. 7:**
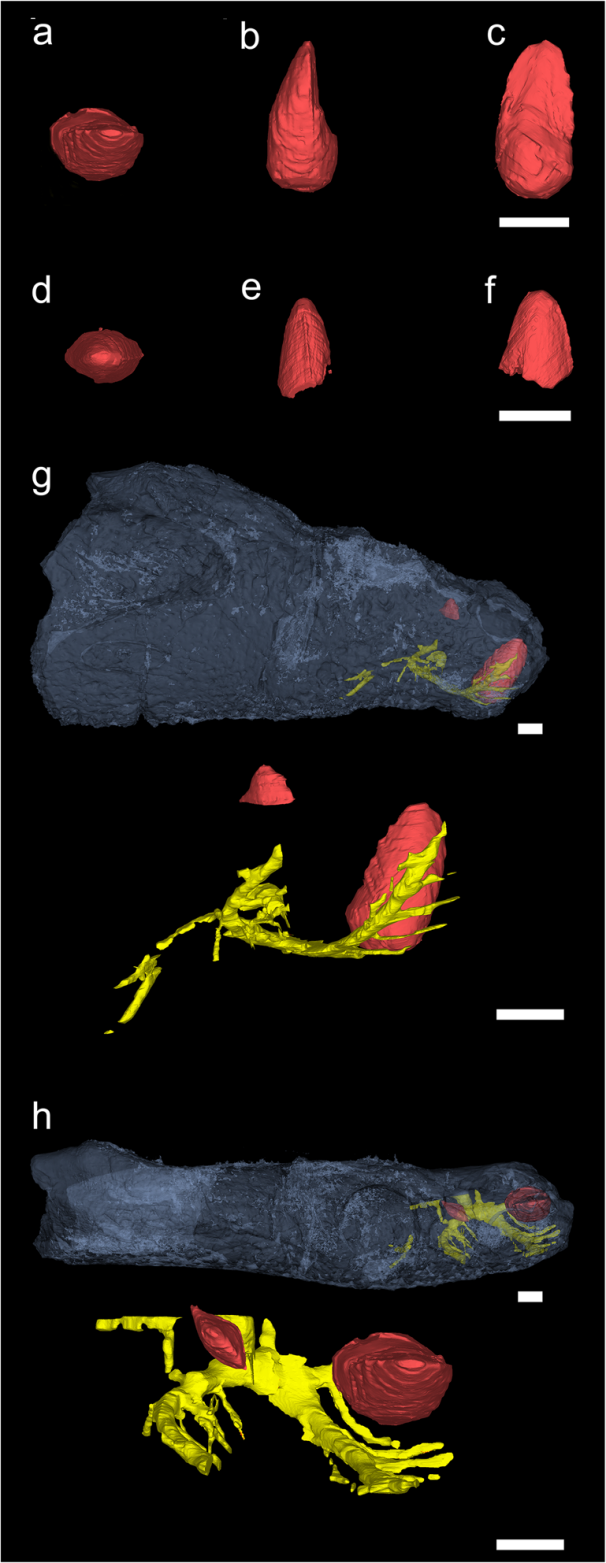
CT scans of the holotype (**a**–**c**, **g**–**h**) and referred (**d**–**f**) specimens. **a**–**c** Replacement teeth of holotype right dentary (NCSM 29059) in **a** occlusal, **b** mesial, and **c** lingual views. **d**–**f** Replacement teeth in paratype left dentary (NCSM 27677) in **d** occlusal, **e** mesial, and **f** lingual views. **g**–**h** CT reconstruction showing vessels (yellow) and replacement teeth (red) of NCSM 29059 in **g** labial (lateral) and **h** occlusal views. Scale bars = 1 cm

**Fig. 8:**
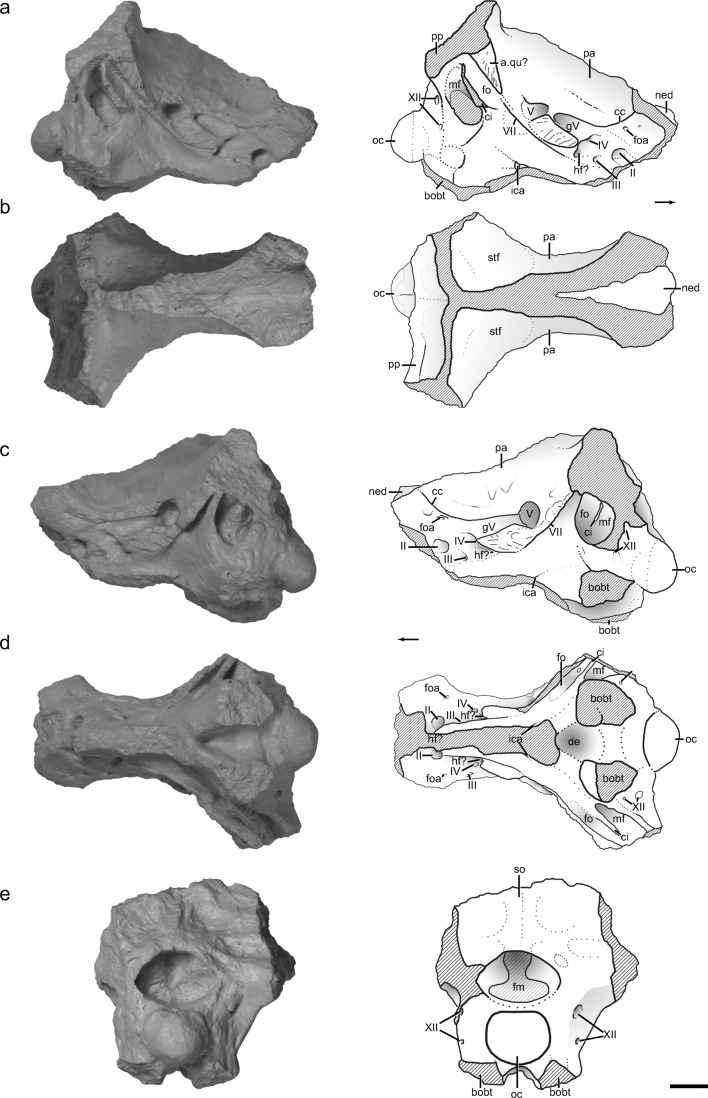
Posterior portion of the skull and braincase (NCSM 27679) in multiple views, with interpretive sketches. **a** Right lateral view, **b** dorsal view, **c** left lateral view, **d** left lateral view, and **e** posterior view. Roman numerals refer to openings for cranial nerves. Other abbreviations: a. qu articulation with quadrate, bobt basal tubera of basioccipital, cc cotylar crest, ci crista interfenestralis, fm foramen magnum, fo fenestra ovalis, foa ophthalmic artery foramen, gV groove for cranial nerve V, hf hypophyseal fenestra, ica entrance of the internal carotid artery, mf metotic, ned natural endocast, oc occipital condyle, pa parietal, pp posterolateral process of the parietal, stf supratemporal fenestra. Arrows indicate anterior direction. Scale bar = 1 cm

Figures 3, 4, 5, 6, 7, and 8, Online Resources 2–8

### Holotype

NCSM 29059–29060, a nearly complete right mandible (Figs. 3, 4, and 5a–d, g–h; Online Resources 2–4) found as two separate, but closely associated specimens in the same horizon. NCSM 29059 is the number assigned to the anterior (tooth-bearing) portion, NCSM 29060 is assigned to the posterior portion. We are certain that they pertain to the same individual, but there is no unambiguous point of contact between the two.

### Locality and horizon

The Downs’ Quarry, at a stratigraphic level slightly above the primary “*Placerias* Quarry” bone level but approximately 1 m below the principal Downs’ Quarry horizon (see stratigraphy and age; Fig. 2). Detrital zircons indicate a maximum depositional age of 219.39 ± 0.16 Ma for this assemblage (Ramezani et al. 2014).

### Paratypes

NCSM 26729, left articular complex (Online Resource 7a–e); NCSM 26730, anterior portion of mandible (Fig. 6a–e, Online Resource 5); NCSM 27677, middle portion of left mandible (Figs. 5e–f, 6f–i); NCSM 27678, left articular complex in two pieces (Online Resource 7f–j); NCSM 29061, right articular complex (Online Resource 8).

### Associated specimens

NCSM 27679, posterior portion of skull and braincase (Fig. 8; Online Resource 6); NCSM 27991, first? sacral vertebral centrum; NCSM 27992, two fused sacral vertebrae.

### Etymology

The genus name comes from the Greek s*yntomi*- meaning “short” and *-prosopus*, meaning “face” in reference to the greatly shortened mandible relative to contemporaneous archosauriforms. The specific epithet *sucherorum* is in honor of Scott (“Major”) and Karen Sucher, who have spent much time and effort over the last 22 years supporting paleontological excavations by ABH and his crews, including participating in and otherwise supporting excavations at the Downs’ Quarry that yielded *Syntomiprosopus*.

### Diagnosis

The presence of denticulated thecodont teeth and an external mandibular fenestra constrain *Syntomiprosopus sucherorum* to at least within Archosauriformes (Nesbitt 2011). *Syntomiprosopus sucherorum* differs from all other archosauromorphs by the following combination of character states: a small external mandibular fenestra is present; an interdigitating suture of the surangular with the dentary; a laterally extensive, rounded, and heavily sculptured surangular ridge on the lateral side of the surangular; a low length-to-depth ratio of the mandible (length:depth ~2.5:1)*; a surangular ridge that completes a 90° arc posteriorly on the lateral side of the surangular*; a dentition restricted to the anterior half of the dentary and consisting of 8 or fewer teeth; anterior and posterior teeth separated by a large (“caniniform”) tooth in the third or fourth position; teeth with a lingual “heel” at their bases; procumbent anterior dentition; uniquely and coarsely sculptured lateral surfaces of the angular, surangular, and dentary*; a well-defined foramen intermedius oralis caudalis bordered medially by the splenial and angular; fine sculpturing consisting of low grooves on the medial surface of the splenial*; well-defined dentary symphysis located on the ventral half of the anteriormost portion of the medial side of the element. Local autapomorphies are identified with an asterisk.

Together with the paratypes, *Syntomiprosopus* is also unique in that it has a variable number of tooth positions anterior and posterior to the identified “caniniform” tooth and high variability in sculpturing of the lateral surfaces of the angular, surangular, and dentary and the medial surface of the splenial.

### Description

#### General description

*Syntomiprosopus* is represented by parts of four different mandibles (two each left and right) representing 2–4 individuals (Figs. 3, 4, 5, 6, and 7), as well as a possibly referable posterior portion of a skull and braincase (Fig. 8). The external surfaces of the mandible bear numerous shallow, densely packed, irregular, fine pits and narrow, arcuate grooves lacking a clear pattern that becomes more rugose posteriorly, especially over the posterior portion of the surangular ridge. The most distinctive feature of *Syntomiprosopus* is its short, robust lower jaw (length ~2.5× depth; ~175 mm:70 mm in the holotype) with as few as four (NCSM 29059) or as many as eight (NCSM 26730) active tooth positions, comprising 2–4 smaller, anterior teeth that may be strongly procumbent, a large alveolus (~12-mm diameter) for the caniniform tooth, and 0–3 “postcanine” alveoli that are in the process of being ontogenetically resorbed in NCSM 26730 (Fig. 6e), a feature described in detail below.

#### Mandible

The mandibles of *Syntomiprosopus* are anteroposteriorly shortened and robust compared to most other Late Triassic reptiles. Their maximum depth increases rapidly posterior to the symphysis and is typically at least twice the symphyseal depth. Throughout the length of the mandible, it is consistently 20 mm or wider, even in gracile specimens (e.g., NCSM 26730). The dentary and splenial appear to be coossified; no suture is visible between the two elements in expected contact areas. The preserved anterior portion comprised of the dentary and splenial forms approximately 60% of the anteroposterior length of the mandible (Fig. 3c). On the lateral surface, a fine patterning of densely packed grooves and pits covers the surface of the bone; this patterning becomes slightly deeper and more irregular dorsally posterolateral to the last alveolus (Figs. 3b and 5b–d). On the lateral surface of the articular complex, these grooves become more elongate and deeper, trending posterodorsally up to and under the extensive dorsolateral edge of the articular and surangular. The external mandibular fenestra is proportionately small compared to most archosauriforms, and only its posteroventral margin is preserved on NCSM 29060 (Fig. 3a). It appears to be nearly circular, but incomplete borders on the dentary and on the postdentary bones do not allow its shape to be determined with certainty. On the medial surface, the holotype (NCSM 29060) preserves an inframeckelian fenestra (= posterior inframeckelian foramen of some workers; e.g., Ford and Benson 2019) that is elliptical, approximately 6.4 mm long by 3.3 mm tall, and located ventral to the internal mandibular fenestra (Fig. 3c). This region is not preserved in NCSM 26730, but the ventral margin of this fenestra may be present on NCSM 27677, which is otherwise broken (Fig. 6h). The posteriormost portion of the internal mandibular fenestra is preserved on the leading edge of the angular on NCSM 29060.

The dentary is twice as dorsoventrally tall posteriorly as it is anteriorly, and generally robust. The anteriormost tooth positions are the most strongly procumbent but become more dorsally directed by the caniniform tooth (Figs. 3 and 6c–e). The posterior tooth positions, if present, are smaller than the more anterior positions and more dorsally directed, as is typical in other archosauriforms. Viewed dorsally, the dentulous portion is U-shaped (Fig. 6e), but the mandibles diverge more widely posterior to the last tooth position such that the lower jaw as a whole was probably nearly as wide as it was anteroposteriorly long (Fig. 4b).

The splenial forms the medial wall of the anterior one-half to two-thirds of the mandible, possibly contributing to the mandibular symphysis. *Syntomiprosopus* preserves a class II (rugose) to class III (interdigitating) mandibular symphysis (Scapino 1981; Holliday and Nesbitt 2013), which are rarer among Triassic reptiles. The symphyseal region is reniform and confined to the anterior portion of the dentary. The most anterior portion of the symphysis is slightly rugose, and the posteroventral portion of the symphysis is much more so, suggesting a stronger connection between the dentaries. Meckelian groove(s) are present in NCSM 26730, trending anteroposteriorly across the ventral one-third of the medial surface of the splenial (Fig. 6c); these grooves appear less extensive in the more robust holotype specimen NCSM 29059 (Fig. 3c). That difference may be that the groove is covered or decreases in length as robusticity increases. In both NCSM 26730 and NCSM 29059, there is a prominent foramen immediately posterior and slightly dorsal to the symphysis in the ventral part of the groove. This may connect internally to a more anterior foramen(?) in the symphysial sutural region; in the holotype, this foramen can be traced to an extensive vascular network associated with tooth positions 1–3 (Fig. 7g–h). In each of NCSM 29059 (Fig. 3c), 26730 (Fig. 6c), and 27677 (Figs. 5e–f and 6h), a very fine vermiform pattern covers the medial surface; this is clearest in NCSM 27677, where the smallest and lightest grooves are interspersed with slightly larger traces indicative of the original vasculature (Figs. 5e–f and 6h).

The angular appears to have extensive dorsoventral and mediolateral expression. It forms the blade-like shape of the posteroventral edge of the mandible (Figs. 3c and 6h) in the articular complex. The sutures with the surangular are difficult to trace; however, the suture with the articular is more evident in NCSM 29060 (Fig. 3c).

The articular forms the glenoid for the articulation with the quadrate, with the medial portion of this glenoid expanding anteriorly with the prearticular (Figs. 3c and 4). A foramen passes dorsoventrally through this expansion (Fig. 3c). There is essentially no retroarticular process (Figs. 3c and 4) because the posterior face of the articular extends ventrally with no posterior expansion, as in some crocodylomorphs (e.g., CM 29894, a specimen referred to *Hesperosuchus agilis*).

The prearticular is most clearly seen on the holotype (NCSM 29060), where it is best exposed in dorsal (Fig. 3b) and posterior views (Fig. 4a), although it is also visible medially (Fig. 3c). It is thin and sheet-like, much taller dorsoventrally posteriorly and tapers rapidly anteriorly. This resembles the condition in some non-mesoeucrocodylian crocodylomorphs (e.g., *Protosuchus*; Dollman et al. 2019).

One of the most striking features of *Syntomiprosopus* is the strongly rugose ridge of bone that forms a 90° arc across the dorsal and posterior margin of the surangular. This surangular ridge extends for more than 25% of the jaw and is typically more than 10 mm thick dorsoventrally, tapering so that the posterior margin, which is oriented more vertically, is somewhat thinner (~10 mm or less anteroposteriorly). It is covered laterally, dorsally, and posteriorly with numerous fine pits and strongly overhangs the lateral side of the jaw. The surface ventral to this overhang is much smoother but pocked by some larger, elliptical pits segregated by thin ridges (best seen in the 3D models—e.g., Online Resources 2 and 4). Ventrally, the finely pitted texture resumes on the lateral surface of the angular and around the posteroventral portion of the jaw. The surangular ridge is also well preserved in NCSM 26729, but the posterior portion is broken off in NCSM 27678 and almost completely destroyed in NCSM 29061. Dorsomedially, the surangular continues anteriorly as a shelf extending along the medial surface of the mandible, though in all specimens described here this shelf is broken and incomplete anteriorly. Dorsolaterally, the surangular has a strongly interdigitating suture with the dentary dorsal to the internal mandibular fenestra, observable in NCSM 27678 and the holotype specimen, NCSM 29059 (Fig. 3c).

#### Dentition

No complete erupted teeth are preserved in any of the specimens described here. However, the size and shape of the alveoli and the preserved replacement teeth preclude assignment of *Syntomiprosopus* to any contemporaneous taxon known only from teeth (e.g., *Uatchitodon*, *Crosbysaurus*, *Kraterokheirodon*, *Krzyzanowskisaurus*). The smaller left jaw (NCSM 26730) preserves at least eight alveoli, four that are mesial to a large caniniform socket and three alveoli distal to it (4-1-3) (Fig. 6e). The larger, holotype right mandible (NCSM 29059) preserves fewer (3-1-0) with the “postcanine” alveoli appearing to be largely resorbed and replaced by dermal bone (Fig. 3b).

The only visible fragments of teeth are in the paratype left dentaries and include the remnants of the base of the caniniform tooth in NCSM 26730 (Fig. 6a–c) and the apical-most tip of a replacement tooth in NCSM 27677 (Fig. 6f–h). The exposed base of the caniniform tooth in NCSM 26730 is nearly circular (~11.9 mm mesial-distal length, 12.1 labiolingual width) with an absolute maximum length (slightly oblique to jaw) of 12.8 mm. The only preserved features of the enamel are a series of fine, apicobasal grooves and ridges, present around the circumference of the tooth but best preserved on the labial side (Fig. 6b). The exposed tip of the emerging tooth crown in NCSM 27677 is relatively round and blunt, with fine denticles that extend basally both mesially and distally from the apex (Fig. 6f–g). Because the crest is broadly arcuate, rather than acutely tipped, we rule out taxa such as *Uatchitodon* (Mitchell et al. 2010) and *Crosbysaurus* (Heckert 2004), which have much more pointed teeth. The denticles are also distinct from those taxa as well as *Kraterokheirodon* (Irmis and Parker 2005), *Krzyzanowskisaurus* (Heckert 2005), *Protecovasaurus* (Heckert 2004), and *Revueltosaurus* (Heckert 2002).

Two replacement teeth are visible in CT data of the anteriormost (most mesial) alveolus of NCSM 29059 (Fig. 7a–c, g–h) and another is visible in CT data for NCSM 27677 (Fig. 7d–f). As reconstructed, the better-preserved tooth is asymmetrical, with a more bulbous labial side and a flatter lingual surface (Fig. 7a–c). It is approximately 23 mm tall (although we cannot differentiate crown from root). Basally, the tooth is ~ 11 mm long and approximately 10 mm wide, but more apically it narrows rapidly and is only 6 mm wide. The mesial and distal edges appear to taper to carinae, and each carina bears multiple small protrusions that could represent large denticles. These denticles are larger on the mesial surface than on the distal edge. This tooth is still in the developmental stage, but its asymmetry, the presence of a basal lingual bulge, and the apparent presence of large denticles are grossly similar in morphology to the denticles of *Kryzanoskisaurus hunti* (e.g., Heckert 2005 figs. 3–6). Anterior to this replacement tooth, another mandible replacement tooth is 12.3-mm distal to mesial length, 10.8-mm labiolingual width, and 23.4-mm height as preserved (Fig. 7g–h). The replacement tooth in the mandible of NCSM 27677 is 9 mm mesiodistally, 6.3 mm in labiolingual width and 10.4-mm height as preserved (Fig. 7d–f).

An emerging replacement tooth in NCSM 27677 is lingual to the large caniniform tooth crown (Fig. 6h). Similarly, there are at least three small pits lingual to posterior teeth on NCSM 26730 (Fig. 6e). This implies that tooth replacement took place by enlargement and lateral migration of the replacement tooth sockets.

#### Posterior portion of the skull

A posterior region of the skull (NCSM 27679; Fig. 8) was closely associated with the paratype partial dentary (NCSM 27677) and posterior half of the hemimandible (NCSM 27678) and collected from the same horizon during the same excavation (2014). There is no unambiguous anatomical evidence linking the posterior region of the skull to the mandibular elements, but we include a description of the specimen because (1) the association of the specimens is in a zone and horizon (Fig. 2b) of the quarry where other taxa are rare, (2) the size of the posterior region of the skull is generally consistent with that of the mandibular elements (Fig. 4b), (3) the preservation is essentially identical and the specimen was recovered close to the paratype, (4) the braincase cannot be referred to any previously known taxon from the *Placerias*/Downs’ Quarry, and (5) the coossification of the elements is consistent with that of the mandibular elements.

The posterior region of the skull is approximately 67 mm long, 48 mm wide, and 48 mm tall as preserved and consists of much of the midline portions of the parietals coossified to the braincase elements (laterosphenoids, basioccipital, prootics, supraoccipital, parabasisphenoid, and partial otooccipitals) (Fig. 8). CT data helps identify a number of openings because these are typically filled with dense material; overall, the contrast between the high-density material within the bone and low-density bone material renders the images difficult to segment and interpret. Thus, nearly the entire description below is based on the external features.

The parietals are fused at the midline as in some early crocodylomorphs (*Dibothrosuchus elaphros* IVPP V 7909) and crocodyliforms. The parietals converge at the midline to form a sagittal crest as in crocodylomorphs (Walker 1990; Nesbitt 2011), but it is not known if the sagittal crest has a small gap between the left and right portions of the crest as in a specimen referred to *Hesperosuchus* (CM 29894; Clark et al. 2001) or the crests are continuous at the midline like *Sphenosuchus actus* (Walker 1990) because of breakage. The posterolateral processes of the parietals diverge from the sagittal crest at an angle near 90° laterally, so that they are in a near coronal plane across the back of the skull (Fig. 8b) that is more similar to crocodyliforms and their close outgroups (e.g., *Almadasuchus gigarii*, Leardi et al. 2020) than to a specimen referred to *Hesperosuchus* (CM 29894; Clark et al. 2001) and other pseudosuchians where the parietals extend posterolaterally. The parietal borders of the supratemporal fenestrae suggest that this opening was large, with a minimum diameter of 16 mm. Anteriorly, the parietals are sheared dorsally, exposing a natural endocast (Fig. 8b).

Collectively, the braincase is well preserved, but it is missing the paroccipital processes, the bases of the basitubera, and the anteroventral portion of the parabasisphenoid (Fig. 8). The basioccipital has a poorly developed neck and the exoccipital portion of the otooccipital forms the dorsolateral portions of the articular surface. The exoccipitals appear to meet on the midline, but the suture between the occipital condyle and the exoccipitals is obliterated. The occipital condyle itself appears slightly ventrally directed at its posterior margin and is slightly wider than tall (15 mm × 12 mm). Ventrally, the basitubera of the basioccipital are well-separated (at least 28 mm wide as preserved) and appear rounded ventrally, although much of their surfaces are abraded. A large lateral ridge of the otooccipital extends laterally so that the metotic opening, crista interfenestralis (=ventral ramus of the opisthotic; Gower 2002), and the fenestra ovalis cannot be seen in posterior view, as in aetosaurs, some rauisuchids, and crocodylomorphs (Gower 2002) and at least one erpetosuchid (Desojo et al. 2011) among pseudosuchians. The openings for cranial nerve XII exit posterolaterally through the lateral ridge, are vertically aligned relative to each other, and the more dorsal opening is larger than the ventral one (Fig. 8e).

Dorsally, the nearly complete supraoccipital forms the posterior portion of the braincase, but the boundaries of this element cannot be discerned because of coossification with the surrounding bones. As a result of this, it is not clear if, or how much, the supraoccipital contributes to the border of the foramen magnum, which is much wider than tall (approx. 18 × 13 mm; Fig. 8e). The nearly vertical supraoccipital bears a midline ridge extending from the dorsal border of the foramen magnum to the dorsal extent of the preserved portion. Lateral to this ridge, several depressions are present. Small depressions on the dorsal border likely represent the contact surface with the proatlas.

The metotic fissure and the fenestra ovalis are well defined, but the morphology within these openings is poorly preserved (Fig. 8a). Likewise, the crista interfenestralis is broken and poorly preserved, but it appears that the structure was anteroposteriorly thin and only separates the metotic fissure and the fenestra ovalis well within a larger opening combining the two; these are character states present in aetosaurs and crocodylomorphs (Gower 2002; Gower and Walker 2002) and at least one erpetosuchid (Desojo et al. 2011). The base of this opening lacks a pendant-shaped ventral end of the crista interfenestralis that extends laterally as in early crocodylomorphs (e.g., *Sphenosuchus acutus*, Walker 1990). However, a structure observed in CT data within the metotic fissure-fenestra ovalis area may be the homologous structure located more medially. The parabasisphenoid is coossified to the other braincase elements so that the sutural contacts are indistinguishable. The parabasisphenoid is highly mediolaterally compressed and anteriorly elongated like that of the rauisuchid *Postosuchus kirkpatricki* (TTUP 9002; Weinbaum 2013) and crocodylomorphs (Walker 1990; Gower 2002), but unlike that of phytosaurs (e.g., Stocker 2010), aetosaurs (e.g., Gower and Walker 2002), and erpetosuchids (Desojo et al. 2011; Nesbitt et al. 2017b). The internal carotid arteries enter the braincase laterally, directly ventral to the opening for cranial nerve V; the exit of the internal carotid arteries cannot be observed because of breakage. Posteriorly, there is a deep fossa between the basitubera of the parabasisphenoid. Although the basipterygoid process is not preserved, the preserved portion of the parabasisphenoid suggests that the structures were located well ventral and anterior to the basitubera like those of rauisuchids (*Postosuchus kirkpatricki*, TTUP 9002) and crocodylomorphs (*Sphenosuchus acutus*, Walker 1990).

Like other elements of the braincase, the boundaries of the prootic are impossible to delimit, so we focus on the features that are consistently part of the prootic. A pronounced ridge (=crista prootica) originates on the anterolateral portion of the opisthotic, trends anteroventrally, and appears to define the contact between the laterosphenoid and the parabasisphenoid. This ridge expands posterolaterally, and the lateral surface is rugose. It is unclear, but this rugose surface may define an articulation surface with the quadrate head, a character state only present in crocodylomorphs (Fig. 8a; Walker 1990; Gower 2002). Just ventral to the ridge, a small foramen within a groove represents the exit of cranial nerve VII. Just dorsal to the ridge, the exit of cranial nerve V opens both laterally and anteriorly. The undivided opening is laterally directed and is circular and larger than all of the cranial nerves in this specimen. The anteriorly directed part is easily traceable in a well-defined and anteriorly widening channel that presumably continues anteriorly to define the contact zone between the prootic and laterosphenoid. On the left side (Fig. 8c), this channel is covered laterally by a bridge of bone, and on the right side, this area is slightly abraded. A rugose area framed by the large ridge and the anterior channel of the exit of cranial nerve V likely represents the attachment location of the epiotic (Holliday and Witmer 2009). A posterolaterally oriented foramen is located dorsal of the large ridge and posterior to the opening of cranial nerve V. Through CT data, it is clear that the exit of cranial nerve VI occurs through the base of the endocranial cavity through the parabasisphenoid as in crocodylomorphs (*Sphenosuchus acutus*, Walker 1990).

The laterosphenoid is fully ossified, preserves well-defined foramina, and is completely coossified with its surrounding elements (parietal dorsally, prootic posteriorly, and parabasisphenoid ventrally). The anteroventral portion of the laterosphenoid contacts the parabasisphenoid, a character state present in aetosaurs and crocodylomorphs (Gower 2002; Gower and Walker 2002), but not in other pseudosuchians. A number of well-defined foramina are present in the anterior portion of the laterosphenoid. The anteriormost opening is the largest of the laterosphenoid openings, and we interpret this as the exit of cranial nerve II. Dorsal to this opening, there are a small set of openings; we interpret the largest one as the ophthalmic artery foramen (see Small 2002). Just posterior to this, there is a ridge that we interpret as the cotylar crest. This ridge stretches from the lateral process (=postorbital process?) of the laterosphenoid to the dorsal border of the anterior channel, originating from the exit of cranial nerve V. Three small openings anterior and ventral to the anterior extent of the anterior channel originating from the exit of cranial nerve V cannot be identified with certainty. We interpret the anteriormost of the three as the exit of cranial nerve III, the most dorsal of the three as the exit of cranial nerve IV, and the most posterior one as the ?hypophyseal fenestra. This hypophyseal fenestra appears to lie on the border with the parabasisphenoid and the laterosphenoid.

The medial border of the vestibule is fully ossified as observed through the foramen magnum. This feature is also present in aetosaurs, rauisuchids, and crocodylomorphs (Gower and Walker 2002).

## Discussion

### Relationships

The presence of an external mandibular fenestra and thecodont, denticulated (serrated) teeth in the holotype and referred jaws of *Syntomiprosopus* constrain these specimens to Archosauriformes based on character optimizations from Nesbitt (2011) and Ezcurra (2016). Unfortunately, there are relatively few characters of the mandible that are phylogenetically informative for Archosauria, and there are not any clearly appropriate phylogenies that incorporate the character sampling of stem archosaurs by Ezcurra (2016) and the early archosaur to crocodyliform sampling in the iterations of Nesbitt’s (2011) dataset into one analysis. Thus, we are hesitant to place this specimen into a current analysis; incorporation of this taxon into a phylogenetic analysis with better character sampling of the mandible (particularly in suchians) must wait for future contributions. We do note that the mandible of *Syntomiprosopus* possesses the type II dentary symphysis, an enlarged “caniniform tooth,” a prearticular that is anteriorly short, and an angular that is well exposed in medial view, character states that have been found in combination within crocodylomorphs (e.g., Dollman et al. 2019), although the symphysis type (Holliday and Nesbitt 2013) and caniniform tooth have a wider distribution among Archosauriformes. The associated posterior portion of the skull and braincase (NCSM 27679) is more phylogenetically informative and may pertain to *Syntomiprosopus* based on its similar size, preservation, and proximity to the paratype jaw, but this association is not definitive in a quarry known for producing large numbers of disarticulated specimens. The skull segment preserves several crocodylomorph character states—a sagittal crest formed by fused parietals, posterior edge of the parietals in a near coronal plane, a laterosphenoid-parabasisphenoid contact, and a possible prootic-quadrate contact (Clark et al. 2001, 2004; Clark and Sues 2002; Nesbitt 2011; Leardi et al. 2017). Yet, the lack of character states present in the crocodylomorph *Sphenosuchus acutus* confine this specimen to an earlier diverging crocodylomorph. These character states include the absence of a deep fossa or fenestra within the basioccipital on the ventral surface, the entrance of the internal carotids is located more dorsally in the plesiomorphic position for pseudosuchians, lack of any large fossa in the parabasisphenoid, and the seeming lack of modifications of the metotic region and crista fenestralis present in *Sphenosuchus acutus* + crocodyliforms. Unfortunately, the distribution of these character states is not well understood at the base of Crocodylomorpha because of the lack of well-preserved braincases.

Given the incomplete nature of NCSM 27679, and the ambiguity of its taxonomic association vis-a-vis the holotype and referred mandibles of *Syntomiprosopus*, we have not scored the taxon into a phylogenetic analysis. Rather, we simply note that it preserves several crocodylomorph features suggesting that *Syntomiprosopus* may actually represent an early diverging crocodylomorph sampling a unique set of crocodylomorph characters rather than an early-diverging archosauriform. If this is the case, face shortening in crocodylomorphs appears to be present at the onset of their diversification and is later repeated by crocodyliform descendants.

### Variation

Although these specimens could represent as few as two individuals, there is still considerable variation present, even on key features of the dentition, splenial, and ornamentation that we use to diagnose *Syntomiprosopus*. Of the tooth-bearing specimens, NCSM 26730 (a left; Fig. 5a–d) is clearly the most gracile, and the holotype (NCSM 29059-29060, a right) and NCSM 27677 (a left, conceivably the counterpart to the holotype) are more robust. The more robust specimens are proportionately thicker, especially across the ventral margin, which is more rounded, whereas this portion of the jaw is narrower and more ridge-like in NCSM 27630. In both the holotype and NCSM 27677, the external patterning wraps around the ventral margin and is visible in medial view, where it extends onto the surface of the fused dentary and splenial (Figs. 3c and 5h). The Meckelian groove/anterior fenestra is actually shorter on the more robust NCSM 29059 than it is on the more gracile NCSM 26730.

Interestingly, the more gracile NCSM 27630, which is also shorter, preserves more tooth positions, possessing the 4-1-3 pattern, whereas the more robust NCSM 29059 is 3-1-2, but with the posterior two positions almost completely resorbed. Similarly, the relatively robust NCSM 27677, which is broken anterior to the caniniform, preserves no unambiguous posterior tooth positions and, like NCSM 29059, has two blind, nearly resorbed positions, albeit with prominent replacement pits lingual to those positions. This is similar to the condition seen in the Middle Jurassic theropod *Limusaurus*, which eventually loses all of its teeth during ontogeny (Wang et al. 2017). Similarly, although many theropod dinosaurs increase the number of tooth positions during ontogeny (see Choiniere et al. 2013), some tyrannosaurids also lose tooth positions such that adults have lower tooth counts than juveniles (Carr 1999, 2020), as does the Triassic theropod *Coelophysis* (Colbert 1989). Ontogenetic tooth reduction in extant crocodylians is not well studied, but the reduction of “postcanine” teeth in Syntomiprosopus involves reducing more tooth positions than typically seen in modern crocodilians, which seldom lose more than one tooth position per element (Brown et al. 2015).

Although all of the specimens with a preserved splenial preserve some ornamentation of that element, the degree of ornamentation varies. The medial surface of the splenial has well-developed grooves and ridges on the ventral portion stretching from the symphysis posteriorly to near the termination of the bone in NCSM 26730. These ridges are not evident in the holotype or preserved portions of NCSM 27677, but all three bones possess the fine, almost vermiform texture on the more dorsal portion of the medial surface of the splenial.

The surangular ridge is present and rugose on all specimens, but the surface ventral to it is much smoother in the holotype right jaw (NCSM 29060) and more rugose, with larger pits separated by more well-developed ridges, in the left jaw NCSM 26729 and right jaw NCSM 29061. In NCSM 26729, the posterior margin of the ridge is more arcuate as well, with the ventral edge beginning to curl back more anteriorly.

### Convergence

The ongoing exploration of Triassic strata in western North America and elsewhere continues to yield archosauriform taxa that are broadly convergent with, and predate by many millions of years, later dinosaurian taxa. Interestingly, these Triassic antecedents include both early-diverging archosauriforms and taxa within the crown. Non-archosaurian archosauriforms that presage later morphologies include the “horned” *Shringasaurus* (Sengupta et al. 2017), converged upon by ceratopsians and some “horned” theropods, and *Triopticus*, which is converged upon by pachycephalosaurs (Stocker et al. 2016). Furthermore, aetosaurs are superficially similar to ankylosaurs, *Revueltosaurus* and silesaurids have dentitions that are convergent with those of early-diverging ornithischians (Parker et al. 2005; Irmis et al. 2007), and the edentulous skulls of shuvosaurids are converged upon by ornithomimid dinosaurs (Chatterjee 1993; Nesbitt and Norell 2006). *Syntomiprosopus* is intriguing in that it appears to be superficially convergent with the crocodyliform *Simosuchus* from the Upper Cretaceous of Madagascar (e.g., Buckley et al. 2000; Kley et al. 2010), as well as the theropod *Limusaurus* from the Jurassic of China. The convergence with *Simosuchus* occurs primarily in the extreme shortening of the jaw; the dentition and mandibular symphyses of *Syntomiprosopus* and *Simosuchus* are not at all similar. The apparent ontogenetic decrease in active tooth positions is the primary similarity with *Limusaurus*. The anatomy of *Syntomiprosopus* suggests that the radiation of Triassic archosauriforms involved exploration of morphospace occupied later not just by dinosaurs but by unusual short-snouted crocodyliforms as well. As other basins approach the intensity of inspection of the American Southwest, additional surprisingly convergent taxa should be recovered.

## Conclusions

*Syntomiprosopus* represents a new taxon with a morphology that is dramatically different from any contemporaneous relative, regardless of whether it is a non-archosaurian archosauriform or a crocodylomorph. If *Syntomiprosopus* is a non-archosaurian archosauriform, it is a relatively late-surviving taxon coexisting with diverse crown-group archosaurs. If, as the associated posterior portion of the skull suggests, it represents an early diverging crocodylomorph, then it reveals a surprising range of morphological innovation among early members of this clade. This new taxon is another example of a Triassic archosauriform taxon whose general morphology is converged upon by later Mesozoic taxa, in this case the short-snouted crocodyliform *Simosuchus* from the Late Cretaceous of Madagascar. Finally, in spite of the extensive history of collecting vertebrates at the *Placerias*/Downs’ Quarry complex, which is the most diverse nonmarine Triassic tetrapod locality currently known, *Syntomiprosopus* demonstrates that even well-studied localities have the potential to yield surprising new taxa.

## Supplementary Information

ESM 1 (ZIP 222 MB)

ESM 2 (MP4 1.31 mb)

ESM 3 (MP4 1.49 mb)

ESM 4 (MP4 1.33 mb)

ESM 5 (MP4 2.50 mb)

ESM 6 (MP4 719 kb)

## Declarations

### Nomenclature

The nomenclatural acts have been registered in ZooBank, the online registration system for the International Code of Zoological Nomenclature. The ZooBank Life Science Identifiers for the taxa in this publication are urn:lsid:zoobank.org:pub:F031EEF8-A8E2-45B9-A268-D65E80FF0F3B [publication], urn:lsid:zoobank.org:act:6D6053EF-BC5D-4878-8CE7-C399CDD9FC5E [*Syntomiprosopus*], urn:lsid:zoobank.org:act:17BBA292-D616-45CC-9541-A0E07845466A [*Syntomiprosopus sucherorum*].
