# Supplementary material for: A new short-faced archosauriform from the Upper Triassic Placerias/Downs’ quarry complex, Arizona, USA, expands the morphological diversity of the Triassic archosauriform radiation
Source: Naturwissenschaften. 2021 Jul 2;108(4):32. doi: 10.1007/s00114-021-01733-1 (PMC8253714; doi:10.1007/s00114-021-01733-1)
Supplement: Supplementary file 1 — (ZIP 222 MB) [file 114_2021_1733_MOESM1_ESM.zip › ESM_OnlineResources_NAWI-D-20-00313R1_Heckertetal/Online Resources Captions.docx]

**Electronic Supplementary Material—Figures**

**Figure Captions**

**Online Resource 2** Photogrammetrically reconstructed 3D model of the holotype right mandible of *Syntomiprosopus sucherorum* (NCSM 29059-29060) without texture overlay to emphasize morphology. Model is available at <https://skfb.ly/6VUE7>

**Online Resource 3** Photogrammetrically reconstructed 3D model of the anterior portion of the holotype right jaw of *Syntomiprosopus sucherorum* (NCSM 29059) with texture overlay showing the fossil as it appears to the naked eye. Model is available at <https://skfb.ly/6VYoP>

**Online Resource 4** Photogrammetrically reconstructed 3D model of the posterior portion of the holotype right mandible of *Syntomiprosopus sucherorum* (NCSM 29030) with texture overlay showing the fossil as it appears to the naked eye. Model is available at <https://skfb.ly/6VYoM>

**Online Resource 5** Photogrammetrically reconstructed 3D model of NCSM 26730, paratype left jaw of *Syntomiprosopus sucherorum*. The pit evident near the anteroventral margin is a taphonomic artifact. Model is available at <https://skfb.ly/6VTuV>

**Online Resource 6** Photogrammetically reconstructed 3D model of NCSM 27679, the posterior skull and braincase found associated with *Syntomiprosopus* *sucherorum*. Model is available at <https://skfb.ly/6VTvn>

**Online Resource 7** Paratype left posterior mandibles of *Syntomiprosopus sucherorum* gen. et sp. nov., a–e, NCSM 26729 in a, dorsal, b, lateral, c, medial, d, posterior, and e, anterior views; f–j, NCSM 27678 in f, dorsal, g, lateral, h, medial, i, posterior, and j, ventral views. Arrows indicate anterior direction. Scale bar = 1 cm

**Online Resource 8** Paratype right posterior mandible of *Syntomiprosopus sucherorum* gen. et sp. nov., NCSM 29061 in a, medial, b, latera, and c, dorsal views. Arrows indicate anterior direction. Scale bar = 1 cm
