# Supplementary material for: A new short-faced archosauriform from the Upper Triassic Placerias/Downs’ quarry complex, Arizona, USA, expands the morphological diversity of the Triassic archosauriform radiation
Source: Naturwissenschaften. 2021 Jul 2;108(4):32. doi: 10.1007/s00114-021-01733-1 (PMC8253714; doi:10.1007/s00114-021-01733-1)
Supplement: Supplementary file 1 — (ZIP 222 MB) [file 114_2021_1733_MOESM1_ESM.zip › ESM_OnlineResources_NAWI-D-20-00313R1_Heckertetal/OR_1_SupplementaryText.docx]

**Electronic Supplementary Materials**

**A new short-faced archosauriform from the Upper Triassic *Placerias*/Downs’ quarry complex, Arizona, U.S.A., expands the morphological diversity of the Triassic archosauriform radiation**

The Science of Nature

Andrew B. Heckert^1,2^, Sterling J. Nesbitt^2,3^, Michelle R. Stocker^2,3^, Vince P. Schneider^2^, Devin K. Hoffman^2^, Brian W. Zimmer^1^

^1^Appalachian State University, Boone, North Carolina

^2^North Carolina Museum of Natural Sciences, Raleigh, North Carolina

^3^Virginia Polytechnic Institute and State University, Blacksburg, Virginia

Corresponding author: Dr. Andrew B. Heckert, Department of Geological & Environmental Sciences, ASU Box 32067, Appalachian State University, Boone, NC 28607-2067; [heckertab@appstate.edu](mailto:heckertab@appstate.edu); telephone: 828-262-7609; FAX 828-262-6503

ORCIDs:

Heckert: <https://orcid.org/0000-0001-9240-6267>

Nesbitt: <http://orcid.org/0000-0002-7017-1652>

Stocker: <http://orcid.org/0000-0002-6473-8691>

Hoffman: <https://orcid.org/0000-0002-9241-8038>

Zimmer: <https://orcid.org/0000-0001-7845-748X>

**Institutional abbreviations—ASU,** Appalachian State University, Boone, North Carolina, USA; **MNA,** Museum of Northern Arizona, Flagstaff, Arizona, USA; **NCSM,** North Carolina Museum of Natural Sciences, Raleigh, North Carolina, USA; **UCMP,** University of California Museum of Paleontology, Berkeley, California, USA.

**Online Resource 1 Details of Materials and Methods, history of collection of the *Placerias/*Downs quarries, and local and regional stratigraphy**

**Materials and Methods**

*Specimen Preparation*

Most specimens were collected as loose elements and wrapped in tissue and/or aluminum foil. Vinac B-15 (polyvinyl acetate) was sometimes used as a field consolidant. Mechanical preparation at NCSM was conducted largely by washing and removal of some resistant matrix with tungsten carbide needles and brushes, with particularly indurated (concretionary) matrix removed by pneumatic tools, principally “microjacks” by Paleotools. In some specimens (e.g., NCSM 26730) areas of missing bone were filled with Apoxie sculpt to maintain specimen integrity.

*Digital Photography*

We photographed the holotype and referred specimens (Figs. 3–5, 6c-e, 6h-i, 8) with a Canon EOS 7D and/or a Nikon D60. To enhance color contrast of the holotype, we coated the specimen in a thin layer of water-based neutral gray paint (Winsor & Newton Designers Gouache, Neutral Gray #3) that was immediately washed off the specimen after photography; other specimens were photographed uncoated. Furthermore, we highlighted the rugosities of the medial surface of the splenial and the lateral surface of the dentary with ammonium chloride using standard techniques (Teichert 1948; Feldman 1989). Detailed photographs of the dentition (microphotographs in Figures 6a-b, f-g) were obtained on a Keyence VHX-5000 3D microscope with VH-Z20R lens in the Nature Research Center in the NCSM, and we used the Keyence software to obtain the tooth measurements reported here. Photo trimming and editing was accomplished in various versions of Adobe Photoshop.

*CT Reconstruction*

In order to more clearly visualize dentition and vascularity, the mandibles NCSM 29059 and 27677 and the braincase NCSM 27679 were scanned at the Shared Materials Instrumentation Facility at Duke University in July 2017 on a Nikon XTH 225 ST high resolution X-ray computed tomography (CT) scanner. The elements were scanned in three batches (NCSM 29059, 27677, and 27679) at 225 kV, with 2000 projections for the mandibles (NCSM 29059 and 27677) and 4000 projections for the braincase (NCSM 27679) in 360 degrees of rotation. Resulting data were analyzed in Mimics v. 19.0 (Materialise NV; [www.materialise.com](http://www.materialise.com)) as 16-bit TIFF images. The data were analyzed on a 64-bit dual platform Mac-PC workstation with 32 GB RAM. Structures of interest were digitally isolated using the segmentation tools in Mimics. Datasets and files of these elements are available from the Morphosource digital repository (www.morphosource.com). Images based on these scans are in Figure 7.

*Photogrammetry and 3D modeling*

We used a Canon Rebel T2i with a 24mm prime lens to photograph the holotype mandible in two parts (NCSM 29059 and NCSM 29060). Using a turntable, we photographed each specimen in 10˚ increments from multiple angles, such that each specimen model is based on approximately 150 images. The images were masked and uploaded to Agisoft PhotoScan Pro ([www.Agisoft.com](http://www.agisoft.com/)) where they were digitally aligned using the workflow proposed by Mallison and Wings (2014) and optimized using techniques developed by Cultural Heritage Imaging ([www.culturalheritageimaging.org](http://www.culturalheritageimaging.org/)). Measurements made on the scaled models have an accuracy of +/- 0.1 mm. NCSM 29059 and 29060 are hypothesized to be from a single individual (see description), although there appears to be approximately 2-3 mm of bone missing, preventing perfect rearticulation of the actual specimens, still our hypothesis for their fit is provided in Supplementary Figure 1. This figure was created without a texture overlay to emphasize subtle morphology and thus does not preserve the true surface coloration. Supplemental Figures 2 (NCSM 29059) and 3 (NCSM 29060) do include this texture and thus accurately portray the surface appearance of both elements in addition to their proportions. The same is true of the model of the paratype right mandible (NCSM 29030, Supplementary Figure 4) and the referred braincase (NCSM 26730, Supplementary Figure 5). The full models can be viewed and downloaded through SketchFab (see Supplementary Figures 1–5) or by contacting the authors directly.

**History of Study and Diversity of the *Placerias* Quarry**

The *Placerias* Quarry was discovered in 1930 by C.L. Camp while trying to relocate a site previously shown to him by a local cobbler, and the history of excavation at the quarry is well documented (e.g., Camp and Welles 1956; Long et al. 1989; Long and Murry 1995; Parker 2018). The vast majority of the known macrovertebrate assemblage is based on collections made by Camp’s crews in the 1930s and is now housed at the UCMP (e.g., Long and Murry 1995). In 1978-79, the MNA excavated the *Placerias* Quarry and a nearby, slightly stratigraphically higher locality, the Downs’ Quarry, and they were the first to employ screenwashing techniques at these sites. The MNA collections include some larger vertebrates but are the basis for the bulk of the microvertebrate diversity from this locality, and the assertion that the *Placerias*/Downs quarry complex is the most diverse nonmarine Late Triassic assemblage (Jacobs and Murry 1980; Murry 1987; Kaye and Padian 1994). The UCMP re-opened the *Placerias* Quarry in 1989, 1990, and 1992, principally to collect more modern taphonomic data (e.g., Fiorillo and Padian 1993; Fiorillo et al. 2000).

Since that time multiple parties have described fossils from the *Placerias* and/or Downs’ Quarry collections at UCMP and MNA These descriptions include an enigmatic phytosaur from the Downs’ quarry (Hunt and Lucas 1991; Padian 1994; Long and Murry 1995, fig. 22; Lucas et al. 1997, fig. 5) that most closely resembles the holotype of ‘*Paleorhinus’ sawini* (but see Stocker 2010, 2013), dinosauromorphs and early dinosaurs (Lucas et al. 1992; Hunt et al. 1998; Nesbitt et al. 2009; Ezcurra and Brusatte 2011), the venomous archosauriform *Uatchitodon schneideri* (Sues 1996; Mitchell et al. 2010), the diapsid *Palacrodon* (Kligman et al. 2018), drepanosaurids (Renesto et al. 2010), and an early anuran (Stocker et al. 2019). Additionally many papers have used the UCMP and MNA collections for comparisons to other assemblages (e.g., Heckert, 2004; Heckert et al., 2005; Irmis, 2005) or to refer taxa (e.g., Parker, 2018). The totality of the assemblage of both larger vertebrates (e.g., Long and Murry, 1995) and microvertebrates (Kaye and Padian, 1994) is extensive, but we refrain from providing any faunal list because the entire assemblage requires reidentification and using more modern approaches (e.g., apomorphy-based identification, see Nesbitt and Stocker, 2008) and comparisons to the rapidly expanding array of tetrapods documented throughout the Chinle.

From 2010-2015, crews from the NCSM and ASU worked both the *Placerias* (2010-2011) and Downs’ (2010-2015) quarries, primarily targeting larger vertebrates, but employing microvertebrate techniques as well. Personnel from Virginia Tech assisted in 2014-2015. All fossils from our excavations, which included fossils in approximately 140 plaster jackets, numerous smaller blocks, and isolated bones and teeth, are reposited at the NCSM. The bones we describe here were recovered in 2013–2015; no material at UCMP or MNA can be unambiguously referred to the new taxon described here.

**Regional Stratigraphy and Age**

Because the *Placerias*/Downs’ Quarry complex is located in an area with relatively poor rock exposure and subdued topographic relief that is also near the southern margin of Chinle outcrop (Akers 1964; Richard et al. 2002; Fig. 1a) its stratigraphic position has been difficult to ascertain and the matter of some debate (see summary by Lucas et al. 1997). Based on lithologic characteristics of the limited exposures surrounding the quarry complex, most workers agree that it is stratigraphically low in the Chinle Formation, and its position has been variously assigned to either the “lower member” (=Blue Mesa Member) of the Chinle unit (Camp and Welles 1956; Jacobs and Murry 1980; Kaye and Padian 1994; Long and Murry 1995; Fiorillo et al. 2000; Irmis 2005; Parker and Martz 2011) or the stratigraphically lower Bluewater Creek Member (Formation of the Chinle Group *sensu* Lucas et al. 1997; Heckert and Lucas 1997, 2003; Heckert et al. 2005) (Fig. 2a). Recent work involving one of us (ABH) used near-surface geophysical techniques to show that the coarse-grained Shinarump Formation does not lie close to the surface, and that the *Placerias* Quarry lies above at least 50 m of strata with similar geophysical characteristics to the exposed mudstone-dominated interval (Bagley et al. 2013). This suggests that the quarry is not near the bottom of the Chinle Formation, or at least does not directly overlie the Shinarump Formation, and thus either lies higher in the Bluewater Creek than hypothesized by Lucas et al. (1997) or else in the Blue Mesa Member.

Recently, Ramezani et al. (2014) obtained a maximum depositional age (MDA) of 219.39±0.16 Ma for detrital zircons they recovered while visiting the NCSM-ASU excavation of the quarry complex in 2013. This age agrees well with numerical estimates obtained from the base of the Blue Mesa Member elsewhere (Heckert et al. 2009; Irmis et al. 2011), although Ramezani et al. (2014) correlated this horizon to the Jasper Forest Bed of the Sonsela Member (Fig. 2a), which yielded a similar maximum depositional age in PEFO, a correlation followed by Parker (2018), but not by Kent et al. (2018) or Marsh et al. (2019), who considered the MDA of the Sonsela to be 216.6 Ma. Instead, Marsh et al. (2019) postulated that some strata assigned to the Blue Mesa Member in New Mexico are actually better referred to the Sonsela Member in New Mexico, but that strata with a similar MDA in Arizona are best assigned to the Blue Mesa Member. This is in part because of paleomagnetic correlations showing that the beds dated to ~218-219 Ma in New Mexico occur in a zone of dominantly reversed polarity, whereas the Sonsela was deposited during an interval of predominantly normal polarity with an onset of ~218–216 Ma in the Petrified Forest National Park, depending on particular age models (Rasmussen et al. 2020; figs. 4–5). At present there is no magnetostratigraphic data from the *Placerias*/Downs’ Quarry complex, but the available lithological, stratigraphic, and geophysical data favor placement of the quarry either high in the Bluewater Creek or else in the Blue Mesa Member (Fig. 2a). We note that one of us (ABH) has unpublished data of LA-ICP-MS U-Pb analyses of detrital zircons from outcrops to the east and stratigraphically slightly higher than the *Placerias* Quarry that yielded an age estimate of 219.22 Ma (+2.3/-2.54 Ma). These are less precise than, but otherwise support, the Ramezani et al. (2014) MDA. In summary, the MDA of 219.32±0.12 Ma provided by Ramezani et al. (2014) is the most precise estimate of the maximum age of the *Placerias*/Downs’ Quarry complex, but the stratigraphic position of these sites within the Chinle Formation remains poorly resolved.

The stratigraphic confusion surrounding the *Placerias*-Downs Quarry complex is unfortunate, because the rich assemblage from the quarry includes almost every taxon considered an index taxon of the Adamanian faunachron or holochronozone (e.g., Lucas and Hunt, 1993; Lucas et al., 1997; Heckert and Lucas, 1997, 2006; Parker and Martz, 2011; Martz and Parker, 2018). Thus, the assemblage has the potential to constrain the first or last appearance datum (FAD or LAD) of the many taxa there as well as to provide a better stratigraphic position for the numerous taxa, such as *Camposaurus arizonensis* and including *Syntomiprosopus sucherorum* gen. et. sp. nov., known only from the quarry complex.

**Local stratigraphy**

In spite of the importance of the *Placerias*/Downs’ quarries, including detailed work on assemblages of microvertebrates (Kaye and Padian 1994) and taphonomy (Fiorillo and Padian 1993; Fiorillo et al. 2000), there is no published stratigraphic column of the quarry complex. Camp and Welles (1956) described two fossiliferous levels at the *Placerias* Quarry, an upper one with a more typical Chinle assemblage of phytosaurs and temnospondyls, separated by ~0.6 m from the primary, lower *Placerias*-dominated bonebed, which is richer and yields more terrestrial animals such as dicynodonts, aetosaurs, and paracrocodylomorphs. Jacobs and Murry (1980) reported that the section was even more fossiliferous than indicated by Camp and Welles (1956), with additional bones below a gypsum layer beneath the primary bonebed, large bones reasonably abundant throughout the section, and smaller bones (the microvertebrates described by Kaye and Padian 1994) especially rich in sandstone stringers. Jacobs and Murry (1980: 57) also described the Downs’ Quarry as 72 m east of the *Placerias* Quarry and essentially on top of the low flat, with less well-preserved pockets of both larger and smaller bones present to as much as 80 cm below the surface, small bone for another 30 cm in a “gray bentonite”, 20 cm of a barren “yellow marly bentonite” before reaching a barren carbonate layer that they thought overlay the primary *Placerias* Quarry bonebed by as much as 3 m. Kaye and Padian (1994) and Fiorillo et al. (2000) largely focused on the *Placerias* Quarry and followed the observations of Camp and Welles (1956) and Jacobs and Murry (1980). Lucas et al. (1997) reported, but did not measure, several meters of bentonitic mudrock, with some of it dark, carbonaceous, and shaly, in the wash below the quarry to the north, and considered the Downs’ Quarry to be only ~1.5 m stratigraphically higher than the main *Placerias* quarry levels.

Our observations of the occurrence of vertebrates at the Downs’ Quarry are broadly congruent with those of Jacobs and Murry (1980) and Lucas et al. (1997), although we excavated a rich layer below the carbonate. Figure 2b shows our interpretations of the local stratigraphy, acknowledging that few, if any, of the horizons are truly planar. The NCSM-ASU excavations recovered fossils from multiple stratigraphic levels, termed “Downs’ Quarry low” and “Downs Quarry high” in the field to discriminate position relative to a persistent carbonate bed (Fig. 2b). Generally speaking, the vast majority of fossils, especially the associated and/or well-preserved bones, came from the lower levels, whereas many more isolated, frequently broken fossils, often intimately associated with coprolites, came from the upper levels. The lower horizons may be genetically related, and the uppermost horizon may also include some bone reworked from the lower horizons. Based on elevation and regional dip, we suspect that the lower horizons are stratigraphically equivalent to, and probably contiguous with, the original *Placerias* Quarry bonebed, although we have not excavated below the lowest bone-bearing horizon. The upper horizon is clearly correlative to the original Downs’ quarry excavations as described by Jacobs and Murry (1980).

At the NCSM-ASU excavations, the stratigraphically lowest horizon includes abundant bones, principally osteoderms and other postcrania of the aetosaur *Desmatosuchus* (e.g., NCSM 26643, a large lateral cervical osteoderm), as well as locally rich pockets of intraformational conglomerate with abundant microvertebrates (Fig. 2b). Approximately a meter above this lowest level is another horizon that is lithologically similar, but contains much less bone and is locally barren, and on the east side of the excavations this interval is marked by a thick pedogenic(?) carbonate. Immediately above this level fossils are uncommon, but include isolated phytosaur postcrania and much, if not all, of the new taxon described here, often associated with coprolites. The strata above this horizon correspond to the original Downs’ Quarry as excavated by the MNA and yields more scattered bones, dominated by incomplete phytosaur skull, jaw, and postcranial elements, many of them weathered and broken prior to burial in the Triassic Period, but they also include diverse smaller tetrapod bones and abundant coprolites. Our work here broadly supports previous taphonomic interpretations of the *Placerias* Quarry (e.g., Fiorillo et al. 2000; Parker 2018), namely that there is little, if any, articulation, but that there is some indication of specimen association in the lower levels. As seen in previous studies, the lowest level is the richest, the bone distribution in the uppermost fossiliferous level is extremely patchy, and there is at least one concretionary horizon (of variable thickness) above the main (lowest) bone level (e.g., Figure 2). The assemblage from the more recent excavations remains under study, but the lower horizon yields many osteoderms and other bones of the aetosaur *Desmatosuchus* (e.g., NCSM 26643, a large lateral cervical osteoderm), incomplete osteoderms of other aetosaurs, including *Calyptosuchus* (NCSM 28188) and cf. *Tecovasuchus* (NCSM 29598), teeth we assign to *Crosbysaurus* (e.g., NCSM 27322-27327) and an array of other tetrapods, including a distal humerus of a dicynodont, presumably *Placerias* (NCsM 26644). The upper horizon yields a more diverse, but fragmentary assemblage including numerous phytosaur bones, osteoderms we tentatively assign to *Calyptosuchus* sp. (e.g., NCSM 25894) and a wide-bodied cf. *Tecovasuchus*-like form (NCSM 25889), the proximal right femur of an allokotosaur (NCSM 26009) and many other bones requiring more study.

**REFERENCES**

Akers JP (1964) Geology and ground water in the central part of Apache County, Arizona US Geological Survey Water Supply Paper 1771:1-107

Bagley CT, Heckert AB, Marshall ST (2013) Using electrical resistivity surveys to determine the stratigraphic position of the Upper Triassic *Placerias* quarry, east-central Arizona. Geological Society of America Abstracts with Programs 45:61

Camp CL, Welles SP (1956) Triassic dicynodont reptiles. Memoirs of the University of California 13:255-348

Cultural Heritage Imaging <http://culturalheritageimaging.org/Technologies/Photogrammetry/>

Ezcurra MD, Brusatte SL (2011) Taxonomic and phylogenetic reassessment of the early neotheropod dinosaur *Camposaurus arizonensis* from the Late Triassic of North America. Palaeontology 54:763-772

Feldmann RM (1989) Whitening fossils for photographic purposes. Paleontological Society Special Paper 4: 342-346

Fiorillo AR, Padian K (1993) Taphonomy of the Late Triassic *Placerias* quarry (Petrified Forest Member, Chinle Formation) of eastern Arizona. New Mexico Museum of Natural History and Science Bulletin 3:133-134

Fiorillo AR, Padian K, Musikasinthorn C (2000) Taphonomy and depositional setting of the *Placerias* quarry (Chinle Formation: Late Triassic, Arizona). Palaios 15:373-386.

Heckert AB (2004) Late Triassic microvertebrates from the lower Chinle Group (Otischalkian-Adamanian: Carnian), southwestern U.S.A. New Mexico Museum of Natural History and Science Bulletin 27:1-170

Heckert AB, Lucas SG (1997) Lower Chinle Group (Adamanian: latest Carnian) tetrapod biostratigraphy and biochronology, eastern Arizona and west-central New Mexico Southwest Paleontological Symposium Proceedings 1:11-23

Heckert AB, Lucas SG (2003) Stratigraphy and paleontology of the lower Chinle Group (Adamanian: latest Carnian) in the vicinity of St. Johns, Arizona. New Mexico Geological Society Guidebook 54:281-288

Heckert AB, Lucas SG (2006) Micro- and small vertebrate biostratigraphy and biochronology of the Upper Triassic Chinle Group, southwestern USA New Mexico Museum of Natural History and Science Bulletin 37:94-104

Heckert AB, Lucas SG, Dickinson WR, Mortensen JK (2009) New ID-TIMS U-Pb ages for Chinle Group strata (Upper Triassic) in New Mexico and Arizona, correlation to the Newark Supergroup, and implications for the "long Norian". Geological Society of America Abstracts with Programs 41:123

Heckert AB, Lucas SG, Hunt AP (2005) Triassic vertebrate fossils in Arizona. New Mexico Museum of Natural History and Science Bulletin 29:16-44

Hunt AP, Lucas SG (1991) The *Paleorhinus* biochron and the correlation of the non-marine Upper Triassic of Pangaea. Palaeontology 34:487-501

Hunt AP, Lucas SG, Heckert AB, Sullivan RM, Lockley MG (1998) Late Triassic dinosaurs from the western United States. Geobios 31:511-531

Irmis RB (2005) The vertebrate fauna of the Upper Triassic Chinle Formation in northern Arizona. Mesa Southwest Museum Bulletin 9:63-88

Irmis RB, Mundil R, Martz JW, Parker WG (2011) High-resolution U-Pb ages from the Upper Triassic Chinle Formation (New Mexico, USA) support a diachronous rise of dinosaurs. Earth and Planetary Science Letters 309:258-267. doi:10.1016/j.epsl.2011.07.015

Irmis RB, Parker WG (2005) Unusual tetrapod teeth from the Upper Triassic Chinle Formation, Arizona, USA. Canadian Journal of Earth Science 42:1339-1345

Jacobs LL, Murry PA (1980) The vertebrate community of the Triassic Chinle Formation near St. Johns, Arizona. In: Jacobs LL (ed) Aspects of Vertebrate History. Museum of Northern Arizona, Flagstaff, pp 55-73

Kaye FT, Padian K (1994) Microvertebrates from the *Placerias* quarry: A window on Late Triassic vertebrate diversity in the American Southwest. In: Fraser NC, Sues H-D (eds) In the shadow of dinosaurs: Early Mesozoic tetrapods. Cambridge University Press, Cambridge, pp 171-196

Kent DV et al. (2019) Magnetochronology of the entire Chinle Formation (Norian age) in a scientific drill core from Petrified Forest National Park (Arizona, USA) and implications for regional and global correlations in the Late Triassic Geochemistry, Geophysics, Geosystems 20:4654-4664 doi:10.1029/2019GC008474

Kligman BT, Marsh AD, Parker WG (2018) First records of the diapsid *Palacrodon* from the Norian, Late Triassic Chinle Formation of Arizona, and their biogeographic implications. Acta Palaeontologica Polonica 63:117-127

Long RA, Lucas SG, Hunt AP, McCrea RT (1989) Charles Camp: collecting Late Triassic vertebrates in the American Southwest during the 1920’s and 1930’s. In: Lucas SG, Hunt AP (eds) Dawn of the Age of Dinosaurs in the American Southwest. New Mexico Museum of Natural History, Albuquerque, pp 65-71

Long RA, Murry PA (1995) Late Triassic (Carnian and Norian) tetrapods from the southwestern United States. New Mexico Museum of Natural History and Science Bulletin 4:254 p

Lucas SG (1998) Global Triassic tetrapod biostratigraphy and biochronology Palaeogeography, Palaeoclimatology, Palaeoecology 143:347-384

Lucas SG, Hunt AP (1993) Tetrapod biochronology of the Chinle Group (Upper Triassic), western United States New Mexico Museum of Natural History and Science Bulletin 3:327-329

Lucas SG, Hunt AP, Long RA (1992) The oldest dinosaurs. Naturwissenschaften 79:171-172

Lucas SG, Heckert AB, Hunt AP (1997) Lithostratigraphy and biostratigraphic significance of the *Placerias* quarry, east-central Arizona. Neues Jahrbuch für Geologie und Paläontologie Abhandlungen 203:23-46

Mallison H, Wings O (2014) Photogrammetry in paleontology—a practical guide. Journal of Paleontological Techniques 12:1-31

Marsh AD, Parker WG, Stockli DF, Martz JW (2019) Regional correlation of the Sonsela Member (Upper Triassic Chinle Formation) and detrital U-Pb zircon data from the Sonsela Sandstone bed near the Sonsela Buttes, northeastern Arizona, USA, support the presence of a distributive fluvial system. Geosphere 15:1128-1139. doi:10.1130/GES02004.1

Martz JW, Parker WG (2017) Revised formulation of the Late Triassic land vertebrate "faunachrons" of western North America: Recommendations for codifying nascent systems of vertebrate biochronology. In: Zeigler KE, Parker WG (eds) Terrestrial Depositional Systems: Deciphering complexities through multiple stratigraphic methods. Elsevier, Amsterdam, pp 39-125

Mitchell JS, Heckert AB, Sues H-D (2010) Grooves to tubes: Evolution of the venom delivery system in a Late Triassic "reptile". Naturwissenschaften 97:1117-1121 doi:10.1007/s00114-010-0729-0

Murry PA (1987) New reptiles from the Upper Triassic Chinle Formation of Arizona. Journal of Paleontology Memoir 61:773-786

Nesbitt SJ, Stocker MR (2008) The vertebrate assemblage of the Late Triassic Canjilon quarry (northern New Mexico, USA), and the importance of apomorphy-based assemblage comparisons Journal of Vertebrate Paleontology 28:1063-1072

Nesbitt SJ, Irmis RB, Parker WG, Smith ND, Turner AH, Rowe T (2009) Hindlimb osteology and distribution of basal dinosauromorphs from the Late Triassic of North America. Journal of Vertebrate Paleontology 29:498-516. doi:doi:10.1671/039.029.0218

Padian K (1994) What were the tempo and mode of evolutionary change in the Late Triassic to Middle Jurassic? In: Fraser NC, Sues H-D (eds) In the shadow of the dinosaurs: Early Mesozoic tetrapods. Cambridge University Press, Cambridge, pp 401-407

Parker WG (2018) Redescription of *Calyptosuchus* (*Stagonolepis*) *wellesi* (Archosauria: Pseudosuchia: Aetosauria) from the Late Triassic of the Southwestern United States with a discussion of genera in vertebrate paleontology. PeerJ 6:e4291. doi:10.7717/peerj.4291

Parker, W.G., and Martz, J.W. 2011. The Late Triassic (Norian) Adamanian-Revueltian tetrapod faunal transition in the Chinle Formation of Petrified Forest National Park, Arizona. *Earth and Environmental Science Transactions of the Royal Society of Edinburgh,* 101(Special Issue 3-4):231-260.

Ramezani J, Fastovsky DE, Bowring SA (2014) Revised chronostratigraphy of the Lower Chinle Formation strata in Arizona and New Mexico (USA): High-precision U-PB geochronological constraints on the Late Triassic evolution of dinosaurs. American Journal of Science 314:981-1008. doi:10.2475/06.2014.01J.

Rasmussen C et al. (2020) U-Pb zircon geochronology and depositional age models for the Upper Triassic Chinle Formation (Petrified Forest National Park, Arizona, USA): Implications for Late Triassic paleoecological and paleoenvironmental change GSA Bulletin in press doi:10.1130/B35485.1

Renesto S, Spielmann JA, Lucas SG (2010) The oldest records of drepanosaurids (Reptilia, Diapsida) from the *Placerias* quarry, Arizona, USA and the stratigraphic range of the Drepanosauridae. Neues Jahrbuch für Geologie und Paläontologie Abhandlungen 252:315-325

Richard SM, Reynolds SJ, Spencer JE, Pearthree PA (2002) Digital graphics files for the Geologic Map of Arizona, a representation of Arizona Geological Survey Map 35, v. 1.0, 1.0 edn. Arizona Geological Survey

Stocker, MR (2010) A new taxon of phytosaur (Archosauria: Pseudosuchia) from the Late Triassic (Norian) Sonsela Member (Chinle Formation) in Arizona, and a critical reevaluation of *Leptosuchus* Case, 1922. Palaeontology 53:997–1022

Stocker, MR (2013) Contextualizing vertebrate faunal dynamics: new perspectives from the Triassic and Eocene of Western North America. Ph.D. dissertation, University of Texas at Austin, Austin, Texas, 317 pp

Stocker MR, Nesbitt SJ, Kligman BT, Paluh DJ, Marsh AD, Blackburn DC, Parker WG (2019) The earliest equatorial record of frogs from the Late Triassic of Arizona. Biology Letters 15:20180922. doi:10.1098/rsbl.2018.0922

Sues H-D (1996) A reptilian tooth with apparent venom canals from the Chinle Group (Upper Triassic) of Arizona. Journal of Vertebrate Paleontology 16:571-572

Teichert C (1948) A simple device for coating fossils with ammonium chloride. Journal of Paleontology 22:102-104
