# Supplementary figures and images for: A new short-faced archosauriform from the Upper Triassic Placerias/Downs’ quarry complex, Arizona, USA, expands the morphological diversity of the Triassic archosauriform radiation
Source: Naturwissenschaften. 2021 Jul 2;108(4):32. doi: 10.1007/s00114-021-01733-1 (PMC8253714; doi:10.1007/s00114-021-01733-1)

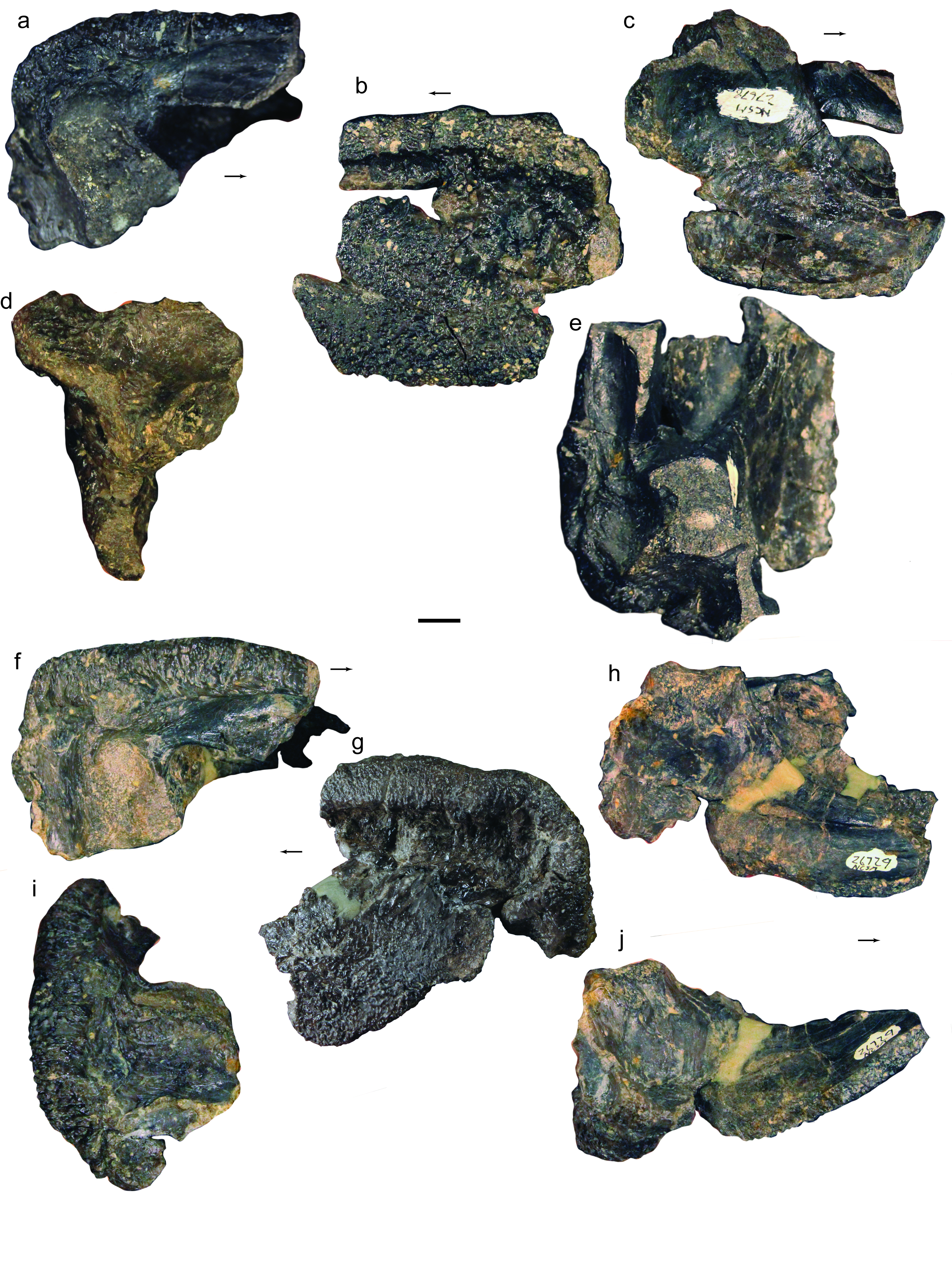

Supplement: Supplementary file 1 — (ZIP 222 MB) [file 114_2021_1733_MOESM1_ESM.zip › ESM_OnlineResources_NAWI-D-20-00313R1_Heckertetal/OR_7_NCSM26729NCSM27678Paratypes.tif]

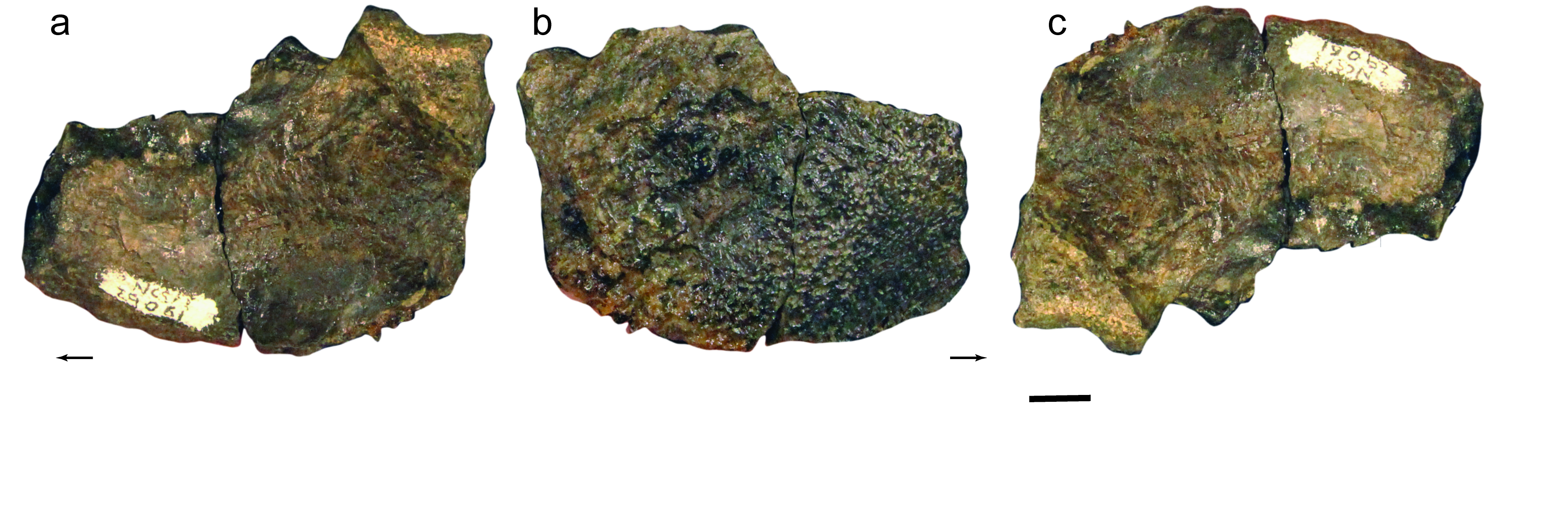

Supplement: Supplementary file 1 — (ZIP 222 MB) [file 114_2021_1733_MOESM1_ESM.zip › ESM_OnlineResources_NAWI-D-20-00313R1_Heckertetal/OR_8_NCSM29061Paratype.tif]
